# Fibrosis‐Guided Ablation in Patients With Atrial Fibrillation: A Meta‐Analysis of Randomized Controlled Trials

**DOI:** 10.1111/jce.16723

**Published:** 2025-06-04

**Authors:** Ahmed Salih, Aman Sutaria, Zeinab Montaser, Tony Pun Magar, Gehad El Ashal, Sheref Zaghloul, Alen Jiji Tom, Mahmood Ahmad, Antonio Creta, Hussam Ali, Sergio Barra, Michal Farkowski, Riccardo Cappato, Rui Providencia

**Affiliations:** ^1^ School of Medicine Imperial College London London UK; ^2^ University College London London UK; ^3^ Nasr City Hospital For Health Insurance Cairo Egypt; ^4^ Oxford Health NHS Foundation Trust Oxford UK; ^5^ Ashford and St Peter's Hospitals NHS Foundation Trust Chertsey Surrey UK; ^6^ Royal Berkshire Hospital, Royal Berkshire NHS Foundation Trust Reading UK; ^7^ Hillingdon Hospital, Hillingdon Hospitals NHS Foundation Trust London UK; ^8^ Department of Cardiology Royal Free Hospital, Royal Free London NHS Foundation Trust London UK; ^9^ Institute of Health Informatics Research University College London London UK; ^10^ Barts Heart Centre, St Bartholomew's Hospital London UK; ^11^ Arrhythmia and Clinical Electrophysiology Center IRCCS, MultiMedica Sesto San Giovanni, Milan Italy; ^12^ Department of Cardiology Hospital da Luz Arrábida Vila Nova de Gaia Portugal; ^13^ Department of Cardiology Ministry of Interior and Administration National Medical Institute Warsaw Poland

**Keywords:** arrhythmia, catheter ablation, mapping, scar, tachycardia

## Abstract

Ablation of fibrotic atrial regions has been suggested to improve the results of atrial fibrillation (AF) catheter ablation. We aimed to evaluate the efficacy and safety of fibrosis‐guided ablation in addition to pulmonary vein isolation (PVI) among AF patients undergoing ablation through a systematic review of randomized controlled trials. The review protocol was registered on PROSPERO (CRD42024561077). Database searches were conducted on EMBASE and MEDLINE until 6th September 2024. Freedom from atrial arrhythmia (including AF and/or atrial tachycardia) and periprocedural complications were the main outcomes. Twelve trials (total of 3,066 patients) were included in the analysis. Ten studies utilized three‐dimensional electroanatomic voltage mapping, and two used magnetic resonance imaging (MRI) to map atrial fibrosis. Compared to PVI, adjunctive fibrosis‐guided ablation significantly improved freedom from atrial arrhythmia (risk ratio [RR] 1.13; 95% confidence interval [CI] 1.04 − 1.23; *p* = 0.004; I² = 35%). This benefit was seen in persistent AF (RR 1.13; 95% CI 1.01 − 1.25; *p* = 0.03), but not paroxysmal AF (RR 1.16; 95% CI 0.83 − 1.61; *p* = 0.20). Only low‐voltage area ablation showed improved freedom from atrial arrhythmias (RR 1.17; 95% CI 1.06 − 1.28 *vs*. RR 1.03; 95% CI 0.80 − 1.32 using MRI‐voltage detection). A numerically, but nonsignificant, higher rate of periprocedural complications was observed with fibrosis‐guided ablation (4.4% *vs*. 2.8%; RR 1.44; 95% CI 0.82–2.56; *p* = 0.18) driven by the results of the DECAAF‐II trial. Fibrosis‐guided ablation, targeting low‐voltage areas on electroanatomic mapping, may be an effective adjunctive target to PVI for improving AF freedom, particularly for persistent AF. However, this approach poses safety concerns.

## 1 Introduction

Atrial fibrillation (AF) represents the most common arrhythmia encountered in clinical practice worldwide, conferring an elevated risk of stroke, heart failure, and mortality [1, 2, 3]. Achieving and maintaining sinus rhythm in AF remains a significant challenge [4]. Pulmonary vein isolation (PVI) is the mainstay of catheter ablation therapy, aiming at sustaining sinus rhythm by electrically isolating the pulmonary veins [4]. Contemporary guidelines and consensus documents have redefined AF from a rhythm disturbance to a composite disease entity encompassing arrhythmia and correlated atrial cardiomyopathy [5]. Nevertheless, ablation procedures for both persistent and paroxysmal AF are associated with the recurrence of atrial arrhythmia, with persistent AF recurrence reaching up to 50% and paroxysmal AF recurrence between 20% and 30% following a single ablation procedures [6, 7, 8].

Various strategies have been investigated to augment clinical outcomes, including further ablation sites beyond just PVI [4]. However, the STAR‐AF II study demonstrated no additional benefits beyond those already known from linear lesions and the ablation of complex fractionated electrograms [9]. Emerging evidence has shown potential association between atrial fibrosis and the re‐entrant circuits perpetuating AF, suggesting fibrotic regions as new potential ablation targets [10]. Low‐voltage areas (LVA) are also known to be strongly associated with AF recurrence post‐catheter ablation in AF [11]. Despite the potential benefits of targeting this surrogate marker of atrial fibrosis, the efficacy and safety of this approach is uncertain, with recent investigations yielding divergent results [12, 13, 14].

Prior meta‐analyses have pooled outcomes for LVA ablation [15, 16] but failed to include other methods for guiding ablation of fibrotic atrial regions, such as late gadolinium‐enhancement cardiac magnetic resonance imaging (MRI) [17]. Furthermore, new trials on LVA ablation have been recently published and not included in previous evidence syntheses [14].

To address these gaps, we conducted a comprehensive systematic review and meta‐analysis of randomized controlled trials (RCTs) to evaluate the efficacy and safety outcomes of PVI plus fibrosis‐guided ablation versus PVI in AF patients.

## 2 Methodology

This systematic review and meta‐analysis were performed and reported following the Cochrane Collaboration Handbook for Systematic Reviews of Interventions and the Preferred Reporting Items for Systematic Reviews and Meta‐Analysis (PRISMA) Statement [18]. The prospective meta‐analysis protocol was registered on the International Prospective Register of Systematic Reviews (PROSPERO; CRD42024561077).

PICO question: *“In patients undergoing catheter ablation for AF, does PVI plus adjunctive fibrosis‐guided ablation make a difference in procedural efficacy and safety when compared to PVI?”*

### 2.1 Database Search

We performed searches on MEDLINE and EMBASE from inception to 6th September 2024. The following search strings were utilized: “atrial fibrillation”, “catheter ablation”, “fibrosis” and “randomized controlled trial”. The full search strategy is given in Supplementary Table 1. Reference lists of possible eligibility studies were also reviewed.

### 2.2 Eligibility Criteria

Studies were considered eligible for inclusion if they were (1) Randomized Controlled Trials; (2) Comparing PVI plus adjunctive fibrosis‐guided ablation versus ablation not targeting fibrotic regions; (3) Enrolled patients with any AF type (paroxysmal, persistent, or long‐standing persistent); (5) Reported prespecified efficacy or safety outcomes of interest (6) and follow‐up duration of at least 12 months. Exclusion criteria include studies that lack comparator arms, non‐randomized and nonhuman studies.

The main outcome of interest was freedom from atrial arrhythmias after a 3‐month blanking period. Atrial arrhythmia recurrence was defined as the presence of either AF and/or any form of sustained atrial tachycardia lasting for more than 30 s. The need for repeat ablation procedures, recurrence of atrial tachycardia and atrial flutter, time to first arrhythmia recurrence, total procedural time, ablation time, and periprocedural complications were also analyzed. A composite of all complications, as reported by the authors, was extracted from each study. Furthermore, major periprocedural complications as defined by *Cappato* and colleagues were also assessed [19]. These included death, cardiac tamponade, sepsis, femoral pseudo‐aneurysm, arteriovenous fistulae, atrioesophageal fistulae, stroke, and pulmonary vein stenosis, those requiring intervention or additional hospital stay.

### 2.3 Study Selection and Data Extraction

All studies identified in our search were screened using the titles and abstracts in Covidence [20]. Each article was independently screened by at least two authors (A.S., Z.H., T.M.), and any discrepancies were resolved through discussion with the senior author (R.P.). Any article identified as having the potential to fulfil our inclusion criteria underwent full‐text evaluation (A.S., Z.H., T.M.) with a senior author resolving conflicts (R.P.). For RCTs deemed eligible, the following relevant data was extracted including: study design, follow‐up duration, ablation technique and criteria for defining atrial fibrosis, characteristics of participants, and ablation outcomes.

### 2.4 Statistical Analysis

For binary endpoints, such as freedom from atrial arrhythmia and periprocedural complications, risk ratios (RR) were used, and the associated 95% confidence interval (CI) was used as the measure of effect size. For continuous endpoints, including procedural and ablation time, weighted mean differences were used to combine results. Time to event was also pooled using hazard ratio (HR) with the associated standard error (SE). Mantel‐Haenszel random‐effects model was used to analyze all the outcomes. All tests were two‐tailed, and a *p*‐value of < 0.05 was considered statistically significant. Heterogeneity was assessed using Cochrane's Q statistic and Higgins and Thompson's I² statistic, with a *p*‐value of ≤ 0.05 indicating statistical significance. Heterogeneity variance was calculated using the restricted maximum likelihood estimator.

We followed the recommendations for thresholds in the Cochrane Handbook for Systematic Reviews of Interventions [21]: 0% to 40%: might not be important; 30% to 60%: may represent moderate heterogeneity; 50% to 90%: may represent substantial heterogeneity; and 75% to 100%: may represent considerable heterogeneity.

We assessed publication bias and other reporting biases by visual inspection of funnel plots for primary outcomes when at least 10 trials were included [21]. Using visual assessment of the asymmetry of the funnel plot we will assess the risk of reporting bias.

The Cochrane Collaboration Risk of Bias tool (RoB‐1) was used to assess the risk of bias in the included trials [22], with the following domains being assessed: random sequence generation, allocation concealment, blinding of participants and personnel, blinding of outcome assessment, incomplete outcome data, selective outcome reporting, & other bias (e.g. evidence of prospective trial registration). These were assessed and judged as high, low, or unclear risk of bias by two review authors (A.S. & R.P.) who independently assessed the risk of bias for each included study. Disagreements were resolved by consultation with a third review author (A.C.).

We the used GRADE system to assess the quality of the body of evidence associated with the selected outcomes in our review [23]. The GRADE approach appraises the quality of a body of evidence based on the extent to which one can be confident that an estimate of effect or association reflects the item being assessed. The quality measure of a body of evidence considers within‐study risk of bias, the directness of the evidence, heterogeneity of the data, precision of effect estimates, and risk of publication bias. The decision to downgrade certainty of evidence resulted from a consensus between two review authors (A.S. and R.P.), and whenever needed a third review author (A.C.) intervened. We added a footnote to the ‘Summary of findings’ table to explain which domains were taken into account in the decision.

The Number Needed to Treat (NNT) was calculated. This was estimated as the reciprocal of the absolute risk difference for the particular outcome between treated subjects and the control group [24].

$$\mathrm{NNT}=\frac{1}{{\mathrm{Absolute Risk}}_{\mathrm{Control Group}}-{\mathrm{Absolute Risk}}_{\mathrm{Treatment Group}}}$$

Additionally, several sensitivity analyses were conducted to see the effect of study factors including: AF patients with baseline atrial fibrosis, AF subtypes (paroxysmal or persistent), method of detecting atrial fibrosis (three‐dimension (3D) mapping or cardiac MRI), ablation technique quality, atrial fibrosis threshold, control group ablation strategy, single *vs.* multicenter RCTs and study year. Sensitivity analyses were only performed for conditions fulfilled by at least two studies. Each study ablation technique was evaluated by the authors and divided into three levels: Exceptional quality ablation (1) required both loss of pacing capture in the fibrotic region and conduction block assessed if linear lesions were deployed; Good quality ablation (2) application of just one of the above‐mentioned tests; Other (3) ‐ none of them.

Calculations and graphics were performed using R version 4.3.4 and the “meta” extension package [25, 26].

## 3 Results

**Figure 1:**
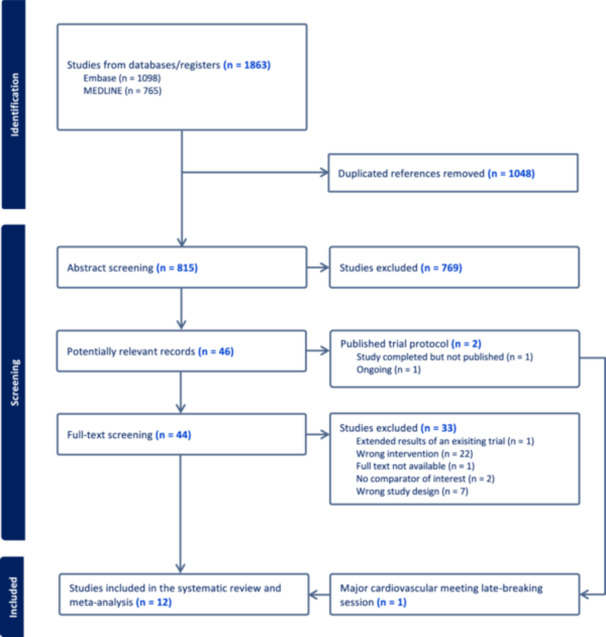
PRISMA flowchart.

Of the 815 potential articles screened, 46 were selected for full‐text review. Among these, 11 randomized controlled trials were identified for analysis [12, 13, 14, 17, 27, 28, 29, 30, 31, 32, 33]. Two trial protocols were also identified in the search [34, 35], of which one is currently ongoing, COAST‐AF (Supplementary Table 2). The other (SUPPRESS‐AF) was presented at a late‐breaking session of a major cardiovascular meeting, European Society of Cardiology 2024 [36], and the results were included, bringing the total included studies to 12 (Figure 1). Supplementary Table 3 provides details on studies excluded following full‐text review.

The population for this meta‐analysis comprised a total of 3,066 patients: 1,529 receiving PVI plus adjunctive fibrosis‐guided ablation and 1,537 treated with PVI and no ablation of fibrosis. Out of the 3,066 patients, 2,301 were male (75.0%), with an overall mean age of 64.3 years. The mean left atrial (LA) dimension was 42.0 mm, and the average duration of known AF pre‐procedure was 28.0 months. Baseline data in the population and the design of the selected trials are summarized in Tables 1 and 2.

**Table 1:** Baseline characteristics of the included trials.

Study	Study design	Participants	Sample Size, *n*		Persistent AF, *n*		Male, *n*		Age (years), mean or median		Hypertension, *n*		Diabetes, *n*	
			Conventional	Fibrosis‐guided	Conventional	Fibrosis‐guided	Conventional	Fibrosis‐guided	Conventional	Fibrosis‐guided	Conventional	Fibrosis‐guided	Conventional	Fibrosis‐guided
STABLE‐SR‐II [14]	Multicenter, single‐blinded, randomized trial	Persistent AF or long‐standing persistent AF	142	134	142 (100%)	134 (100%)	99 (69%)	88 (66%)	60.4 ± 9.6	60.6 ± 9.4	58 (41%)	65 (49%)	14 (11%)	11 (8%)
Kaiser et al. [27]	Single‐center, randomized trial	Persistent AF or long‐standing persistent AF	50	50	50 (100%)	50 (100%)	36 (72%)	33 (66%)	67.5 ± 10.8	65.2 ± 8.9	55 (73%)	44 (59%)		
STABLE‐SR‐III [12]	Multicenter, single‐blind, randomized trial	Paroxysmal AF 65 to 80 years	219	219	0 (0%)	0 (0%)	108 (49%)	111 (51%)	70.7 ± 4.1	70.2 ± 4.7	142 (65%)	130 (59%)	43 (19%)	32 (15%)
STABLE‐SR [29]	Multicenter, single‐blind, randomized trial	Nonparoxysmal AF	115	114	115 (100%)	114 (100%)	84 (73.7%)	92 (81%)	57.6 ± 8.4	57.1 ± 9.5	63 (55%)	62 (54%)	10 (9%)	8 (7%)
Kircher et al. [28]	Single‐center, open‐label, randomized trial	Paroxysmal or persistent AF	62	62	25 (40%)	36 (58%)	41 (66%)	36 (58%)	63 ± 9	62 ± 10	50 (81%)	49 (79%)		
VOLCANO [32]	Single‐center, open‐label, randomized trial	Paroxysmal AF with LVA	32	30	0 (0%)	0 (0%)	9 (28%)	9 (30%)	74.7 ± 8.0	75.3 ± 7.2	16 (50%)	20 (67%)	6 (19%)	10 (33%)
ERASE‐AF [13]	Multicenter, open‐label, randomized trial with blinded endpoint assessment	Persistent AF	163	161	163 (100%)	161 (100%)	104 (64%)	112 (70%)	66 ± 10	60 ± 10	137 (84%)	130 (81%)	40 (25%)	40 (25%)
Hwang et al. [27]	Single‐center randomized trial	Persistent AF	25	25	25 (100%)	25 (100%)	23 (92%)	20 (80%)	57.9 ± 9.8	58.8 ± 9.3	11 (44%)	14 (56%)	4 (16%)	5 (20%)
Wang et al. [33]	Single‐center randomized trial	Persistent AF	60	64	60 (100%)	64(100%)	35 (58%)	41 (64%)	62.2 ± 5.7	62.8 ± 10.5	24 (40%)	26 (41%)		
ALICIA [17]	Multicenter, open‐label, randomized trial	Paroxysmal or persistent, or longstanding persistent AF	76	79	32 (42%)	38 (49%)	55 (72%)	56 (71%)	59 ± 10	58 ± 10	45 (59%)	38 (48%)	9 (12%)	5 (6%)
DECAAF II [28]	Multicenter, open‐label, randomized trial	Persistent AF	422	421	422 (100%)	421 (100%)	333 (79%)	332 (79%)	63.2, 57.1‐68.8	62.2, 57.0–68.2	247 (59%)	247 (59%)	45 (11%)	40 (10%)
SUPPRESS‐AF [36]	Multicenter, open‐label, randomized trial	Persistent AF with left atrial LVAs > 5 cm^3^	171	170	171 (100%)	170 (100%)	89 (52%)	85 (50%)	74.7 ± 6.1	73.8 ± 6.8	125 (74%)	117(68%)	35 (21%)	42 (25%)

Study	AF duration (months), mean or median		AAD use, *n*		LAD (mm), mean		CHADSVASC score, mean or median		LVEF (%), mean		Follow‐up
	Conventional	Fibrosis‐guided	Conventional	Fibrosis‐guided	Conventional	Fibrosis‐guided	Conventional	Fibrosis‐guided	Conventional	Fibrosis‐guided	
STABLE‐SR‐II [14]	6.0, 1.0–12.0	6.0, 2.0–15.5	55 (39%)	60 (45%)	42.5 ± 5.3	41.4 ± 5.9			62.1 ± 6.8	61.3 ± 9.2	18 months
Kaiser et al. [27]			31 (62%)	30 (60%)			2.55 ± 1.74	2.67 ± 1.58	50.3 ± 9.6	53.4 ± 5.2	12 months
STABLE‐SR‐III [12]	14, 4.0–48.0	24.0, 6.0–48.0	65 (30%)		38.8 ± 5.4	38.8 ± 5.4	2.5 ± 1.0	2.3 ± 0.8	62.4 ± 5.4	62.4 ± 5.3	12 months
STABLE‐SR [29]	15.9 ± 33	18.9 ± 29	42 (37%)	35 (31%)	40.7 ± 4.8	41.1 ± 5.3			62.0 ± 6.6	61.8 ± 7.7	18 months
Kircher et al. [28]	60, 36–111	54, 24–87	26 (42%)	30 (48%)	42 ± 6	43 ± 6	2, 1–3	2, 1–3	61 ± 7	59 ± 9	12 months
VOLCANO [32]	5, 2–23	4, 2–14	19 (59%)	20 (67%)	38 ± 5	40 ± 6	3.3 ± 1.3	3.6 ± 1.2	65 ± 10	64 ± 14	12 months
ERASE‐AF [13]	31, 12–77	31, 11–77	17 (11%)	17 (11%)	45 ± 6	45 ± 7	3, 2–4	3, 2–4	54 ± 11	53 ± 12	12 months
Hwang et al. [27]	27.1 ± 19.2	21.4 ± 26.2			50 ± 6	49 ± 4	1.4 ± 1.4	1.8 ± 1.4	57.9 ± 10.0	59.4 ± 11.4	13 months
Wang et al. [33]	32.8 ± 15.8	40.8 ± 38.1			45.2 ± 3.3	45.1 ± 4.5			63.5 ± 5.1	64.6 ± 5.7	14 months
ALICIA [17]	54 ± 71	59 ± 65	71 (93%)	77 (98%)	42 ± 7	43 ± 6	1.3 ± 1.1	1.2 ± 1.2	56 ± 8	56 ± 9.6	15 months
DECAAF II [28]			250 (59%)	240 (57%)							12–18 months
SUPPRESS‐AF [36]			13 (8%)	9 (5%)	43.6 ± 5.5	44.1 ± 5.4	3.5 ± 1.5	3.4 ± 1.3	57.4 ± 10.4	55.8 ± 10.7	12 months

*Note:* Data are presented as mean ± SD, and median, interquartile range.

Abbreviations: AAD, antiarrhythmic drugs; AF, atrial fibrillation; LAD, left atrial diameter; LVEF, left ventricle ejection fraction.

**Table 2:** Design of the included trials.

Study	Control arm	Experimental arm	Fibrosis detection method	Atrial fibrosis threshold	Location of fibrosis	Ablation technique	Prespecified lesion quality control^a^	Energy source of catheter ablation	Arrythmia follow‐up detection method	Arrythmia endpoint definition
STABLE‐SR‐II [14]	PVI only	PVI + LVA ablation	Electroanatomic mapping system	Low voltage area = 0.1–0.4 mV and transitional zones = 0.4–1.3 mV		Homogenization ablation in the LVAs and defragmentation in the transitional zones	2. Map on sinus > 300 HD points; target AI 500 for anterior, 450 for roof, and 400 for inferior and posterior segments; LVA: target voltage of < 0,1 mV for ablated areas, no info on loss of capture/isolation of LVA; posterior wall box instead of homogenization; no info on conduction block assessed if linear lesion deployed	Radiofrequency	Clinic visits and 24‐h Holter recordings at 3 and 6 months, and 7‐day Holter recordings at 12 months postablation. Subsequent years, 24‐h Holter every 6 months. Additional ECG for symptomatic patients.	AF or atrial tachycardia off AADs after a 3‐month blanking period, each episode > 30 s.
Kaiser et al. [27]	PVI with linear ablation and/or elimination of non‐pulmonary vein triggers	PVI + LVA ablation	Electroanatomic mapping system	< 0.5 mV		Isolation or homogenization of the fibrotic zones with proof of entrance and exit blocks	1. HD map on sinus; target AI 380 at the posterior wall and 480 at the anterior wall; LVA ablation: reduction of electrogram voltage of more than 50% and a loss of pacing capture; conduction block assessed if linear lesion deployed.	Radiofrequency	48 h Holter‐ECG every 3 months. Undocumented symptoms suspicious of arrhythmia were documented by additional external ECG.	AF or atrial tachycardia off AADs after a 3‐month blanking period, each episode > 30 s.
STABLE‐SR‐III [12]	PVI only	PVI + LVA ablation	Electroanatomic mapping system	< 0.5 mV in ≥ 3 adjacent low‐voltage points		LVA homogenization to create dense scar or box isolation	2. Map on sinus, non‐HD mapping with ablation catheter > 150 points; AI 500 for anterior, 400 to 450 for roof, and 350 to 400 for inferior and posterior segments; no info on loss of capture/isolation of LVA; no infomation on conduction block assessed if linear lesion deployed; statement that ablation strategy described previously but no reference available	Radiofrequency	Clinic visits and 24‐h Holter recordings at 3 and 6 months, and 7‐day Holter recordings at 12 months postablation. Subsequent years, 24‐h Holter every 6 months. Additional ECG for symptomatic patients.	Atrial tachyarrhythmias off AADs on ECG documentation during clinical visit or > 30 s during Holter recordings
STABLE‐SR [29]	PVI with stepwise ablation	PVI + cavotricuspid isthmus + LVA ablation	Electroanatomic mapping system	0.1–0.4 mV and complex electrograms within transitional zones (0.4–1.3 mV)		Homogenization of LVA tissue and elimination of complex electrograms in the transitional zones	2. Map on sinus > 500 HD points; no info on AI/LSI for lesion creation, no info on loss of capture/isolation of LVA; conduction block assessed if linear lesion deployed	Radiofrequency	Clinic visits and 24‐h Holter recordings at 1, 3 and 6 months, and 7‐day Holter recordings at 12 months postablation. Subsequent years, 24‐h Holter every 6 months. Additional ECG for symptomatic patients.	AF or atrial tachycardia off AADs after a 3‐month blanking period, each episode > 30 s.
Kircher et al. [28]	PVI with additional linear ablation for persistent AF	PVI + LVA ablation	Electroanatomic mapping system	< 0.5 mV in ≥ 3 adjacent low‐voltage points	LVAs were mainly located at the left atrial posterior wall, at the left atrial roof, and at the inter‐atrial septum	Regional LVA homogenization with additional linear lesions or electrical isolation depending on several factors	1. Non‐HD map on sinus; ablation 35/25 W in power mode, no contact measurement; LVA ablation: loss of pacing capture; conduction block assessed if linear lesion deployed	Radiofrequency	Serial 7 day Holter recordings were obtained at 3, 6, and 12 months and any post‐blanking arrhythmia episode documented outside the Holter recording periods	AF, atrial flutter or atrial tachycardia off AADs after a 3‐month blanking period, each episode > 30 s.
VOLCANO [32]	PVI only	PVI + LVA ablation	Electroanatomic mapping system	< 0.5 mV and covering > 5.0 cm²	LVAs were predominantly observed in the anterior‐septal wall in 92% patients, followed by the roof in 42%, posterior wall in 29%, inferior wall in 11%, and lateral wall in 8%	LVA homogenization	3. Non‐HD map on sinus; ablation 30 W in power mode, no contact measurement; LVA ablation: voltage reduction of > 50%, box isolation confirmand by pacing, no wide anterior wall or septal ablation; no info on conduction block assessment if linear lesion deployed	Radiofrequency and cryoballoon	Routine 12‐lead ECGs and 24‐h ambulatory Holter monitoring was performed 6 and 12 months postablation. Additional ECG for symptomatic patients.	After 3‐month blanking period off AADs: (1) AF or atrial tachycardia on ECG during routine outpatient visit or symptoms (2) AF or atrial tachycardia > 30 s on Holter monitoring.
ERASE‐AF [13]	PVI only	PVI + LVA ablation	Electroanatomic mapping system	< 0.5 mV	The anterior wall shows the highest prevalence (75%), followed by the septum (65%), while the lateral wall had the lowest prevalence (11%)	Isolation of LVA or scarred left atrial tissue	1. Non‐HD map on sinus; ablation 40–45 W in power mode, contact 10–20 grams; LVA ablation: loss of pacing capture; conduction block assessed if linear lesion deployed	Radiofrequency	7‐day ECG recordings were obtained at 3, 6, and 12 months and optional ICM	AF or atrial tachycardia off AADs after a 3‐month blanking period, each episode > 30 s on 7‐day ECG. Atrial arrhythmia documented on any wearable, or implanted ECG device also contributed.
Hwang et al. [27]	PVI only	PVI + LVA ablation	Electroanatomic mapping system	Complex fractionated atrial electrogram within areas of < 0.5 mV		Complete elimination by targeted ablation	3. Map on AF; 30–60 s applications to eliminate CFAE; no info on loss of capture/isolation of LVA; no info on conduction block assessment if linear lesion deployed	Radiofrequency	12‐lead ECG and 24‐h Holter monitoring at 3, 6, and 12 months postablation. ECG and Holter monitoring for symptomatic patients.	AF or atrial tachycardia off AADs after a 3‐month blanking period, each episode > 30 s.
Wang et al. [33]	PVI with stepwise ablation	PVI + LVA ablation	Electroanatomic mapping system	Double and fractionated potentials within areas of < 0.5 mV		Elimination of action potentials within LVA with additional linear ablation in severe atrial disease	1. Non‐HD map on sinus; ablation 30–35 W in power mode; LVA ablation: loss of pacing capture; conduction block assessed if linear lesion deployed	Radiofrequency	12‐lead ECG every 3 months or with symptoms. In all patients 24‐h Holter monitoring was used at 6, 9 and 12 months postablation. Monthly telephone inquiry to evaluate AF recurrence.	Atrial tachyarrhythmias > 30 s (with or without AADs) after 3‐months blanking period
ALICIA [17]	PVI only	PVI + MRI‐guided atrial fibrosis ablation	Delayed‐enhancement CMR	Voxel signal intensity ratio to mean LA blood pool pixel intensity – > 1.20 for native fibrosis and > 1.32 for dense scar		Homogenization or isolation	1. HD map but no info if on sinus; ablation 40/30 W anterior/posterior wall with CF; LVA ablation: reduction of electrogram voltage to < 0,1 mV and a loss of pacing capture; conduction block assessed if linear lesion deployed.	Radiofrequency	12‐lead ECG and 24 Holter recording at 3, 6, and 12 months.	AF or atrial tachycardia off AADs after a 3‐month blanking period, each episode > 30 s.
DECAAF II [28]	PVI only	PVI + MRI‐guided atrial fibrosis ablation	Delayed‐enhancement CMR	Based on Marrek DE‐MRI protocol using dynamic threshold algorithm process enhanced/fibrotic voxels of the LA		Encircle ablation of the fibrosis perimeter and/or completely cover all fibrotic areas with ablation lesions	2. No HD map on sinus; target ablation settings according to local protocol but a minimum of 8–10 s and 10 g of force; LVA ablation: loss of pacing capture; no info on conduction block assessed if linear lesion deployed.	Radiofrequency and cryoballoon	Smartphone ECG device checked at 6 and 12 months follow‐up. Back‐up ambulatory monitoring or 12‐Lead ECG.	Atrial arrhythmia (including atrial tachycardia, atrial flutter, or atrial tachycardia) > 30 s after 3‐month blanking period
SUPPRESS‐AF [36]	PVI only	PVI + LVA ablation	Electroanatomic mapping system	< 0.5 mV		Homogenization and posterior wall isolation for posterior wall LVAs	3. HD map on pacing HD; AI ≥ 350; no info on loss of capture/isolation of LVA; no info on conduction block assessed if linear lesion deployed	Radiofrequency	24‐h Holter ECG monitoring and outpatient 12 lead ECG at 3, 6 and 12 months. Daily home ECG recordings at 6 and 12 months.	Atrial arrhythmia including AF and/or AT lasting ≥ 30 s by 12‐lead ECG or other appropriate tests off AADs.

Abbreviations: AADs, antiarrhythmic drugs; AF, atrial fibrillation; AI, artificial intelligence; DE‐MRI, delayed enhancement magnetic resonance imaging; CMR, cardiac magnetic resonance; ECG, electrocardiogram; HD, high‐density; LA, left atrial; mV, millivoltage; LVA, low‐voltage area; PVI, pulmonary vein isolation.

^a^ 1 indicates exceptional quality ablation, 2 indicates good quality ablation and 3 indicates other.

Ten trials utilized 3D electroanatomic mapping, and two trials used delayed‐enhancement (DE) MRI to guide atrial fibrosis ablation. Two studies were on patients with paroxysmal AF, and seven were on patients with persistent or long‐standing persistent AF. Three studies included patients with both paroxysmal and persistent AF. In most studies, the control group received only PVI ablation, but there were four studies employing PVI with additional ablation strategies not targeting fibrotic regions. These included stepwise and linear ablation (Table 2).

Most studies performed randomization before fibrosis mapping. In the VOLCANO trial, randomization occurred after voltage mapping, and only patients with LVA were included in this analysis [32]. In the SUPPRESS‐AF trial, only patients with confirmed LVA were recruited and subsequently randomized [36]. The DECAAF II trial randomized participants by Utah Stage, using cardiac MRI to stratify the degree of atrial fibrosis [28].

Among studies of electroanatomic mapping, LVA was defined as areas with < 0.5 mV in eight studies and 0.1–0.4 mV in two studies. For the MRI‐guided ablation studies, the DECAAF II trial used the DE‐MRI protocol to quantify left atrial wall enhancement [28]. A dynamic threshold algorithm was applied to identify fibrotic voxels in the LA. These processed DE‐MRI images were integrated with a 3D mapping system to guide ablation. While in the ALICIA trial DE‐MRI images underwent post‐processing using the dedicated ADAS software to generate a 3D reconstruction of the LA with fibrosis information [19]. Fibrosis was identified based on voxel signal intensity, with a signal intensity ratio to mean LA blood pool pixel intensity of > 1.20 for native fibrosis and > 1.32 for dense scar tissue.

The majority of studies used a combination of serial 12‐lead electrocardiogram (ECG) ambulatory, Holter monitoring and symptomatic ECG recording to detect atrial arrhythmia in the follow‐up period. Only DECAAF II used a smartphone‐based ECG device as the primary arrhythmia monitoring method [28]. All studies utilized radiofrequency catheter ablation, while VOLCANO and DECAAF II also used cryoballoon ablation [28, 32]. In regard to the quality of the ablation technique utilized, five studies were considered to be excellent (both loss of pacing capture in the fibrosis area and conduction block assessment), four were good quality (one of the above) and three were other (none).

Supplementary Figure 1 illustrates the risk of bias associated with each trial. Five studies had all domains classified as low risk of bias, and four studies had one domain (i.e., blinding of patients) classified as high risk. Three studies had no high‐risk domains but presented at least one domain classified as unclear risk.

Funnel plots showed absence of publication bias (Supplementary Figure 2).

GRADE assessments are presented in the Summary of Findings Table (Supplementary Table 4), alongside with the rationale for downgrading quality of evidence. Quality of evidence was considered moderate for the primary outcome, freedom from atrial arrhythmia relapse. Quality of evidence was considered very low for the endpoints total procedure time and atrial tachycardia relapse, and low for all remaining outcomes.

### 3.1 Efficacy

**Figure 2:**
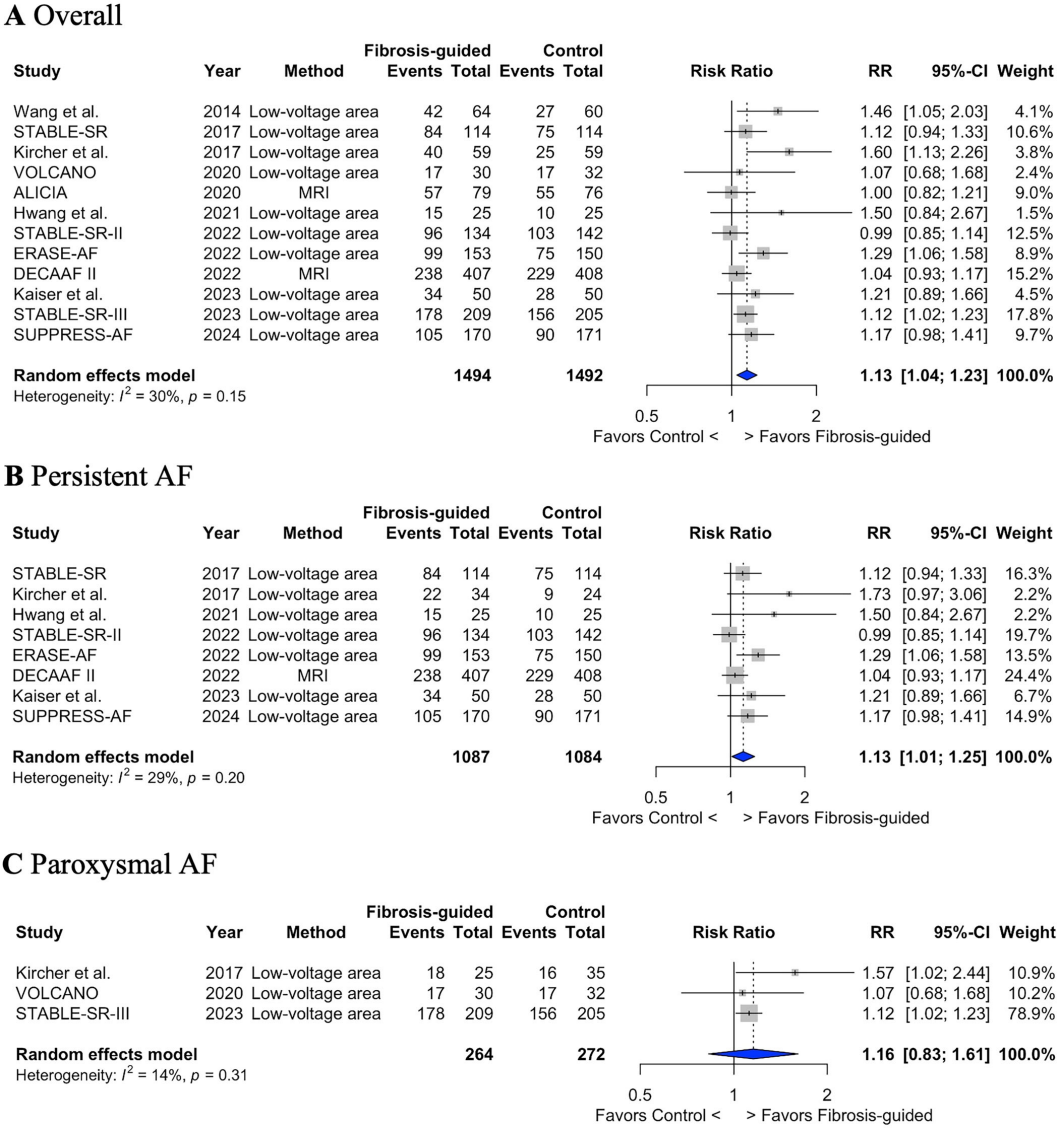
Freedom from atrial arrhythmias. Forest plot illustrating the comparison of freedom from atrial arrhythmia outcomes between fibrosis‐guided and conventional ablation in all patients (A), as well as specifically among persistent (B) and paroxysmal AF patients (C). RR ‐ risk ratio; 95%CI ‐ 95% confidence intervals. A Overall.B Persistent AF. C Paroxysmal AF.

Compared with conventional ablation, fibrosis‐guided ablation improved freedom from atrial arrhythmia (67.3% *vs.* 59.7%; RR 1.13; 95% CI 1.04 − 1.23; *p* = 0.004; I^2^ = 35%; NNT = 13.1 patients to prevent one atrial arrhythmia relapse) (Figure 2A). When considering only patients with confirmed baseline atrial fibrosis (LVA or MRI) a similar improvement in freedom from atrial arrhythmia was observed (66.8% *vs.* 52.5%; RR 1.22; 95% CI 1.11 − 1.33; *p* = 0.002; I^2^ = 0%; NNT = 7.0) (Supplementary Figure 3). In time‐to‐event analysis, adjunctive fibrosis‐guided ablation significantly reduced atrial arrhythmia recurrence (HR 0.79; 95% CI 0.67 − 0.94; *p* = 0.008; I^2^ = 19%) (Supplementary Figure 4). There was also a reduction in the need for repeat ablation procedures (12.1% *vs.* 16.9%; RR 0.72; 95% CI 0.56 − 0.93; *p* = 0.023; I^2^ = 0%; NNT = 20.8) (Supplementary Figure 5). Fibrosis‐guided ablation did not increase the recurrence rates of AT (7.4% *vs.* 8.5%; RR 0.75; 95% CI 0.37 − 1.55; *p* = 0.38; I^2^ = 60%) or atrial flutter (7.8% *vs.* 6.4%; RR 1.22; 95% CI 0.35 − 4.28; *p* = 0.29; I^2^ = 0%) (Supplementary Table 4).

There was a significant improvement in freedom from atrial arrhythmia among patients with persistent AF (63.8% *vs.* 57.1%; RR 1.13; 95% CI 1.01 − 1.25; *p* = 0.0324; I^2^ = 29%; NNT = 15.0) (Figure 2B). However, for patients with paroxysmal AF, freedom from atrial arrhythmias was greater but not statistically significant in the fibrosis‐guided ablation arm (80.7% *vs.* 69.5%; RR 1.16; 95% CI 0.83− 1.61; *p* = 0.20) (Figure 2C).

**Figure 3:**
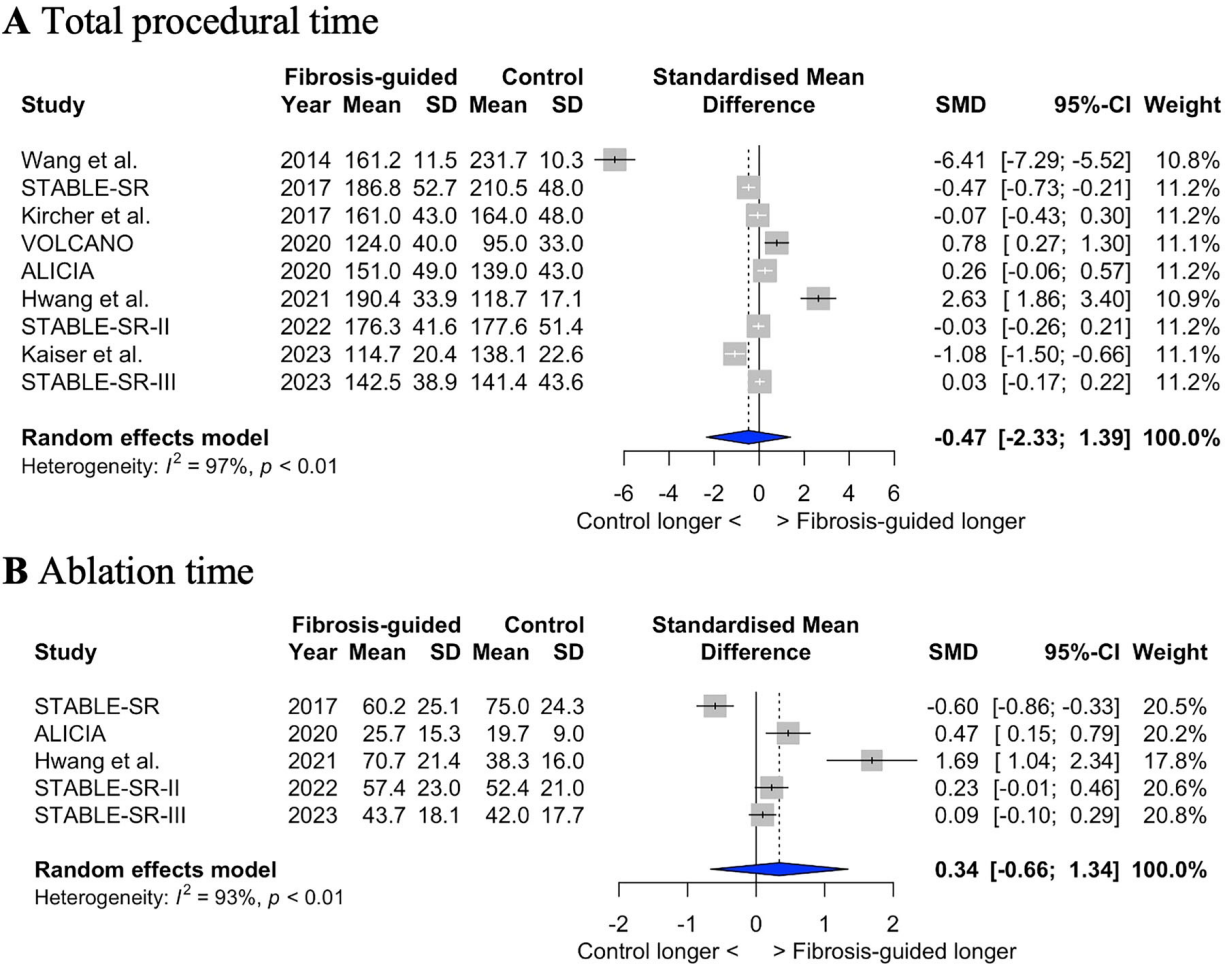
Procedural and ablation time. Forest plots showing the differences in total procedural time (A) and ablation time (B) between fibrosis‐guided and conventional ablation. SMD ‐ standardized mean difference; 95%CI ‐ 95% confidence intervals.

There was no statistically significant difference in total procedural time between the two groups (standardized mean difference −0.47; 95% CI − 2.33 to 1.39; *p* = 0.58; I^2^ = 97%) (Figure 3A). Similarly, there was no difference in total ablation time (standardized mean difference 0.34; 95% CI − 0.66 to 1.34; *p* = 0.40; I^2^ = 93%) (Figure 3B). Similarly, when considering RCTs with PVI only as the control ablation arm there was no difference in either ablation or procedural time (Supplementary Figure 6).

**Figure 4:**
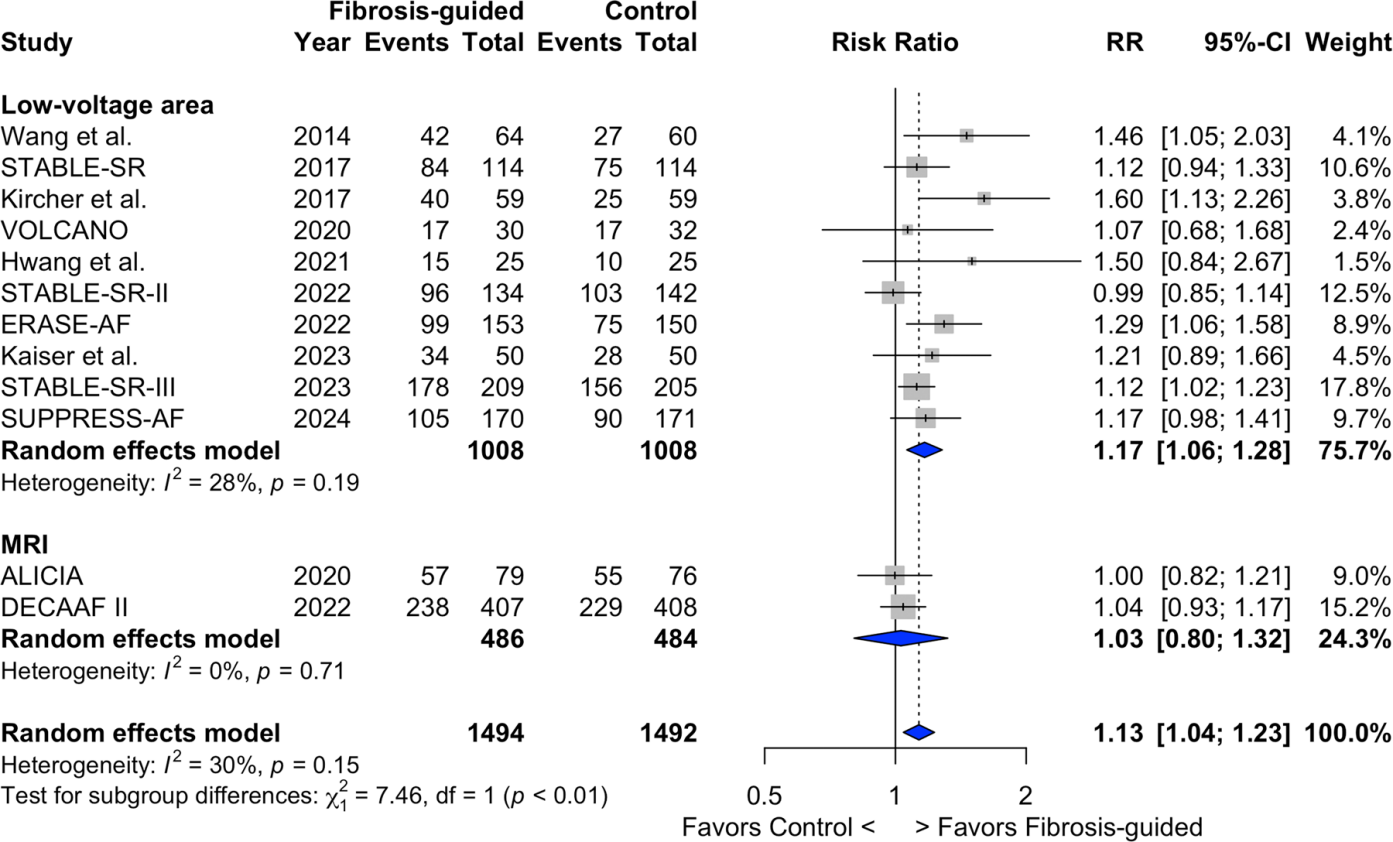
Freedom from atrial arrhythmias based on atrial fibrosis mapping method. Forest plot comparing outcomes between fibrosis‐guided and conventional ablation strategies based on fibrosis mapping method. RR ‐ risk ratio; 95%CI ‐ 95% confidence intervals.

Studies using voltage mapping for LVA‐guided ablation demonstrated statistically significantly improved freedom from atrial arrhythmias (70.4% *vs.* 60.1%; RR 1.17; 95% CI 1.06 − 1.28; I^2^ = 27%; NNT = 9.7) compared to those using DE‐MRI to map cardiac fibrosis (60.7% *vs.* 58.7%; RR 1.03; 95% CI 0.80 − 1.32; I^2^ = 0%) (Subgroup difference *p* < 0.01) (Figure 4).

RCTs were categorized based on the quality of the ablation technique. Studies using exceptional quality ablation techniques demonstrated the highest improvement of freedom from atrial arrhythmia (67.2% vs. 53.2%; RR 1.25; 95% CI 1.00–1.57; I^2^ = 50%). Those employing lower but good‐quality techniques showed a moderate improvement (69.0% vs. 64.8%; RR 1.07; 95% CI 0.98–1.18; I^2^ = 0%). Studies where the ablation quality was either unspecified or deemed lower demonstrated a modest rate of freedom from atrial arrhythmia (60.9% vs. 51.3%; RR 1.18; 95% CI 0.93–1.50; I^2^ = 30%). While studies with higher ablation quality showed numerically greater freedom from arrhythmia, subgroup differences did not reach statistical significance (*p* = 0.09) (Supplementary Figure 7).

We stratified the outcome of freedom from atrial arrhythmias according to the type of ablation approach in the control group: PVI alone or with PVI and additional non‐fibrosis‐guided ablation. The control group type had no statistically significant effect on freedom from atrial arrhythmia with fibrosis‐guided ablation ‐ subgroup difference: RR 1.09 versus RR 1.27, *p* = 0.08 (Supplementary Figure 8). Freedom from atrial arrhythmias was also analyzed based on varying fibrosis low‐voltage definitions across the studies: two trials used a 0.1–0.4 mV threshold, and eight trials used a < 0.5 mV threshold. No statistically significant difference was observed between the two thresholds, subgroup difference: RR 1.04 versus 1.20, *p* = 0.06 (Supplementary Figure 9).

Our sensitivity analysis showed that multicenter RCTs demonstrated a significantly lower magnitude of freedom from atrial arrhythmia improvement than those conducted in a single‐center ‐ subgroup difference: RR 1.09 versus 1.36, *p* < 0.0048 (Supplementary Figure 10).

The pooled freedom from atrial arrhythmia from the five RCTs that were classed as low bias had a significant improvement in freedom from atrial arrhythmia with fibrosis‐guided ablation compared to control (68.9% *vs.* 64.7%; RR 1.08; 95% CI 1.00–1.16; I^2^ = 0%, *p* = 0.048) (Supplementary Figure 11).

Stratifying RCTs by publication date (studies published before 2020 compared to those published after 2020) did not significantly change the outcomes ‐ subgroup difference: RR 1.32 versus 1.09, *p* = 0.11 (Supplementary Figure 12).

### 3.2 Safety

**Figure 5:**
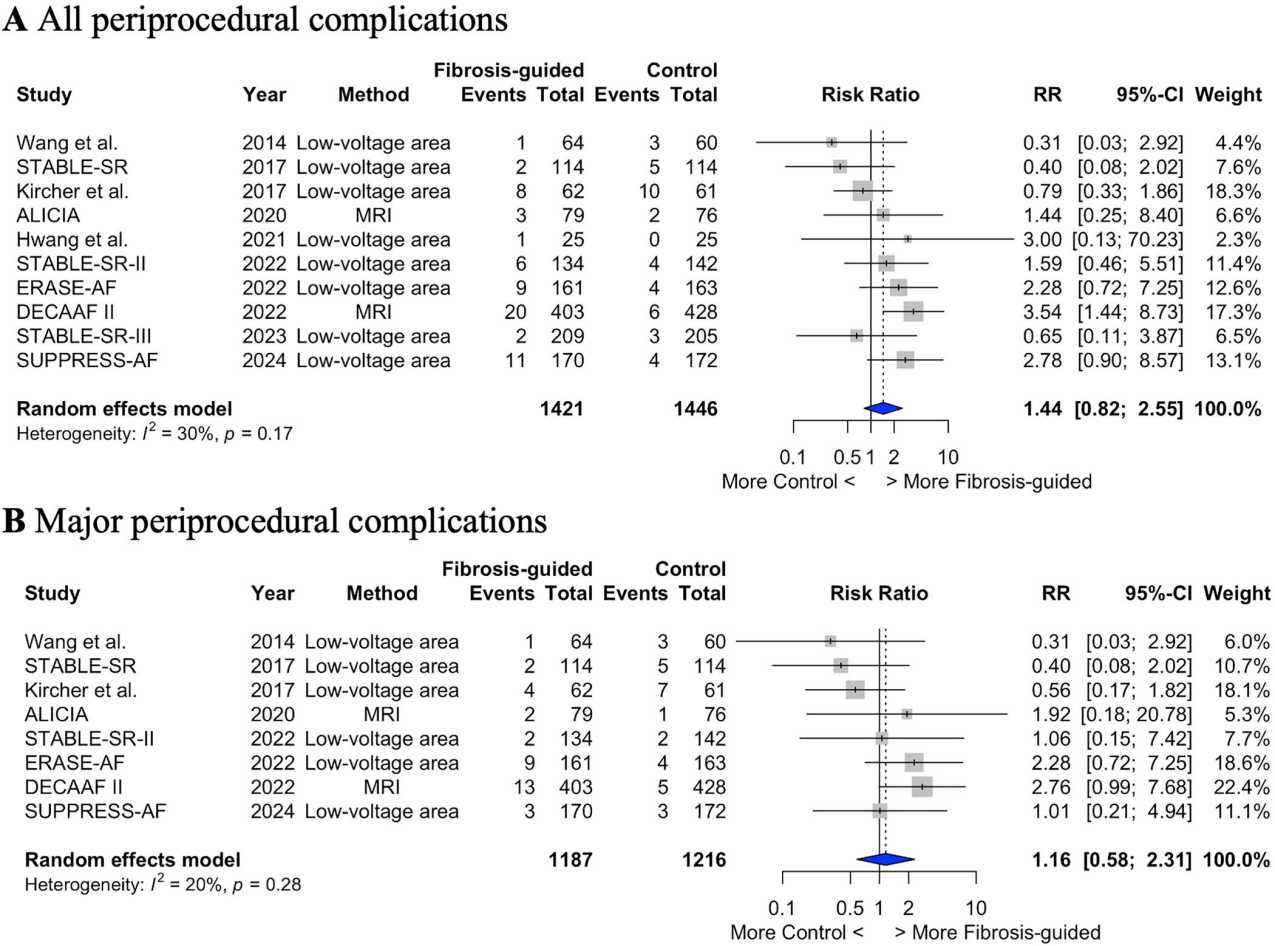
Periprocedural complications. Forest plots comparing periprocedural complications between fibrosis‐guided and conventional ablation strategies. (A) All periprocedural complications and (B) major periprocedural complications. RR ‐ risk ratio; 95%CI ‐ 95% confidence intervals.

The incidences of complications and major adverse events were low. However, there was numerically higher, but nonsignificant, rate of periprocedural complications with the addition of fibrosis‐guided ablation (4.4% *vs.* 2.8%; RR 1.44; 95% CI 0.82–2.56; *p* = 0.18; I^2^ = 30%; NNT = 50.7 patients to cause one additional complication) (Figure 5A). A numerically higher complication rate was still observed after removing trials of cardiac MRI‐based fibrosis ablation, but differences were attenuated (4.3% *vs.* 3.5%; RR 1.19; 95%CI 0.63–2.24; *p* = 0.55; I^2^ = 17%; NNT = 67.4) (Supplementary Figure 13). When considering only major periprocedural complications there were no statistically significant differences between the fibrosis ablation and conventional ablation groups (3.0% *vs.* 2.5%; RR 1.16; 95% CI 0.58–2.31; *p* = 0.62; I^2^ = 20%) (Figure 5B).

## 4 Discussion

The results of this systematic review and meta‐analysis suggest that adding fibrosis‐guided ablation to PVI may significantly improve freedom from atrial arrhythmias when compared to PVI‐based ablation in patients with AF. This benefit was observed for persistent AF while there was a nonsignificant but likely underpowered result for paroxysmal AF. No benefit was observed for cardiac‐MRI guided fibrosis ablation. An observation of higher complication rate requiring further clarification was observed for patients in the fibrosis‐guided ablation group, driven by the cardiac‐MRI guided ablation DECAAF‐II trial. A moderate degree of heterogeneity was found for some analyses, and lack of patients was an issue for some studies, which contributes to some degree of uncertainty in our findings.

Our findings provide some support to the hypothesis that targeting atrial fibrosis, a marker of atrial myopathy and structural remodeling, may enhance the success of AF ablation, albeit with some increment in procedural‐associated risk [37]. The inclusion of only randomized controlled trials provides robustness to these findings. To our knowledge, this is the first meta‐analysis that has compared patients with AF undergoing PVI to any form of adjunctive fibrosis‐guided ablation (i.e. either 3D electroanatomic mapping or DE‐MRI).

Atrial fibrosis ablation is one of a multitude of potential additional extra‐PVI ablation targets, such as linear lesion sets, posterior wall isolation, complex fractionated electrograms, and focal impulse and rotor modulation [4]. Ablation of atrial fibrosis has become increasingly common, with 18.4% of the writing members of the recent European Heart Rhythm Association (EHRA) consensus on AF ablation performing ablation of MRI‐ or voltage mapping‐detected abnormal atrial myocardial areas during redo paroxysmal AF ablation procedures with an additional 13.2% and 31.6% ablating fibrosis during first‐time, and redo ablation of persistent AF, respectively [4]. A key contemporary challenge in fibrosis‐guided ablation is the clinical variability of fibrosis itself. While fibrosis is a recognized indicator of arrhythmogenic substrate, not all fibrotic areas necessarily harbor re‐entrant circuits or contribute directly to AF perpetuation. Hence, the difficulty in distinguishing between fibrotic areas that actively sustain arrhythmias versus those that do not remain a conflicting issue [38]. Prior studies suggest that only specific fibrotic regions, particularly those demonstrating low voltage on electroanatomic mapping, are closely linked to atrial fibrillation pathology [39]. This highlights the complexity of targeting fibrosis as an independent strategy and highlights the need for improved techniques that can accurately identify and treat clinically significant fibrotic areas.

The variability in methods for detecting atrial fibrosis across the studies also presents an important consideration. Two primary techniques used were presented in this meta‐analysis—3D electroanatomic mapping and DE‐MRI. Both techniques are used to define the atrial substrate, albeit using quite different surrogates of atrial fibrosis—bipolar voltage as a marker of atrial fibrosis and late gadolinium signal intensity, respectively. Late gadolinium enhancement MRI offers a noninvasive, comprehensive assessment of fibrotic tissue, providing detailed visualization of the extent and distribution of fibrosis [40]. However, the lack of real‐time feedback during ablation and potential limitations in spatial resolution may explain the nonsignificant improvement in AF freedom observed in this meta‐analysis. In contrast, 3D electroanatomic mapping provides real‐time data on electrical activity, enabling precise targeting of LVAs. Additionally, discrepancies between MRI and endocardial mapping have been reported [41]. This difference may explain the differences in outcomes between 3D electroanatomically‐ and MRI‐guided ablation of supposed fibrotic substrate (Figure 4).

Strategies for ablating atrial fibrosis can also vary across centers and operators, with direct ablation of the entire LVA/homogenization, encircling/box isolation of LVA, and modification of the LVA, being some of the suggested approaches [42]. There is uncertainty on which is the most effective approach to achieve freedom from AF relapse.

Furthermore, methodologic challenges inherent to the strategy of low voltage–guided substrate modification still need to be addressed. Voltage measurements are dependent on rhythm status (AF *vs.* sinus rhythm), size, and configuration of the recording electrodes. Furthermore, voltage parameters indicative of abnormal substrate lack objective definition and low‐voltage cutoffs may vary considerably among different investigators. While a one‐size‐fits‐all voltage cutoff could provide a solution, this does not consider regional variations in atrial wall thickness, nor the nature of the recording electrodes [43].

An additional consideration is the timing of randomization relative to identifying areas of atrial fibrosis. Most of the included RCTs randomized patients before conducting fibrosis mapping, resulting in many patients without low‐voltage areas receiving only standard pulmonary vein isolation despite being in the intervention. This inconsistency may dilute the observed effect of low‐voltage area ablation, as a significant proportion of patients may not benefit from fibrosis‐guided ablation. SUPRESS‐AF [36] stands as a unique investigation, that only randomized participants after performing electroanatomic mapping, only proceeding when LVAs are detected. In this meta‐analysis, when only considering patients with baseline fibrosis a 22% improvement in freedom from atrial arrhythmia was observed.

Another crucial factor in interpreting the results of this meta‐analysis is the variability in the control group ablation strategies. In eight of the included studies, the control group underwent pulmonary vein isolation alone, while in the remaining four studies, additional ablation strategies were permitted [29, 30, 31, 33]. Importantly, our sub‐analysis excluding the four studies with additional lesions beyond PVI in the control group, shows comparable efficacy results.

The observed trend for a potential safety signal with fibrosis‐guided ablation in our review, adds to the safety concerns with cardiac‐MRI guided fibrosis ablation in the DECAAF II trial, where a significantly higher rate of safety outcomes (2.2% vs 0%; *p* = 0.001) was observed, with two deaths and six ischemic strokes observed in the cardiac‐MRI guided fibrosis ablation group (*vs.* none in patients treated with PVI alone) [28].

Despite these important insights, this meta‐analysis is not without limitations. First, the variability in ablation protocols, the use of different energy sources, and differences in the endpoints for fibrosis‐guided ablation across studies may introduce potential bias. Second, the observed heterogeneity, assessed by I^2^, was moderate for the pooled analysis of freedom from atrial arrhythmia, driven by studies of persistent AF patients. Third, the small number of studies on patients with paroxysmal AF limits the conclusions that can be drawn for this group. Fourth, all studies utilized radiofrequency as the source of energy for ablation. Further studies are required for assessing the efficacy and safety of novel technologies, like pulsed‐field ablation, that are now transitioning to clinical practice. Fifth, while arrhythmia recurrence is the standard outcome among the included studies, other significant clinical endpoints, such as AF burden and the persistent to paroxysmal AF conversion rate, were not reported and therefore analyzed in this meta‐analysis. Additionally, due to the low rate of complications, this systematic review lacks statistical power for definitive inferences on the safety of fibrosis‐guided ablation. Finally, while the supposed benefit from the investigated strategy may depend on effective elimination of the arrhythmogenic activity within the targeted fibrotic tissue, it is not possible to exclude that, irrespective of selected ablation targets, benefit is simply related to the excess curative treatment as compared to PV isolation only.

## 5 Conclusion

Fibrosis‐guided ablation, targeting low‐voltage areas on 3D mapping, may be an effective adjunct to PVI in patients under catheter ablation for persistent AF. Ablation of cardiac‐MRI detected fibrosis appears to be of no benefit. Further studies are needed to clarify the efficacy for paroxysmal AF and the potential safety signal associated with LVA‐directed ablation.

## Conflicts of Interest

Professor Rui Providencia has a Research Grant from by Biosense‐Webster for supporting a PhD Fellow on a project on AF mechanisms and genetics; Professor Riccardo Cappato reports grants from Pfizer, Daiichi Sankyo, Boehringer Ingelheim, Johnson and Johnson, grants and personal fees from Boston Scientific, Medtronic, Abbott and Biosense‐Webster. No other conflicts of interest to report.

## Supporting information

AF ablation supporting material 030324.

## Data Availability

No new data were generated or analysed in support of this study. All data were extracted from publications or provided by study authors and made available on the manuscript.
