# Supplementary material for: Fibrosis‐Guided Ablation in Patients With Atrial Fibrillation: A Meta‐Analysis of Randomized Controlled Trials
Source: J Cardiovasc Electrophysiol. 2025 Jun 4;36(8):2025–40. doi: 10.1111/jce.16723 (PMC12337619; doi:10.1111/jce.16723)
Supplement: Supplementary file 1 — AF ablation supporting material 030324. [file JCE-36-2025-s001.docx]

**Supplementary material**

**Supplementary Table 1: Search Strategy for Medline and EMBASE**

| **Search Terms** |
| --- |
| (atrial fibrillation OR AF OR Afib) |
| AND |
| (ablation OR catheter ablation OR pulmonary vein isolation) |
| AND |
| (low-voltage area OR low-voltage OR fibrosis OR fibrotic OR Magnetic Resonance Imaging OR MRI OR substrate OR modification) |
| AND |
| (Randomized Controlled Trials OR RCT OR random* OR trial*) |

**Supplementary Table 2: Trial protocol without published results**

| **Study title** | **Year** | **Registration ID** | **Source** |
| --- | --- | --- | --- |
| Characterization of arrhythmia substrate to ablate persistent atrial fibrillation (COAST-AF): Randomized controlled trial design and rationale. | 2022 | NCT03347227 | https://dx.doi.org/10.1016/j.ahj.2022.08.010 |
| The efficacy and safety of left atrial low-voltage area guided ablation for recurrence prevention compared to pulmonary vein isolation alone in patients with persistent atrial fibrillation trial: Design and rationale (SUPPRESS-AF) | 2021 | UMIN000021831 | https://doi.org/10.1002/clc.23677 |

**Supplementary Table 3: Excluded studies**

| **Study title** | **Year** | **Reason** | **Source** |
| --- | --- | --- | --- |
| Efficacy and safety of targeted focal ablation versus PV isolation assisted by magnetic electroanatomic mapping. | 2003 | Wrong intervention - targeted focal ablation | https://dx.doi.org/10.1046/j.1540-8167.2003.02468.x |
| Substrate modification combined with pulmonary vein isolation improves outcome of catheter ablation in patients with persistent atrial fibrillation: a prospective randomized comparison. | 2006 | Wrong intervention - linear atrial ablation | https://dx.doi.org/10.1093/eurheartj/ehl093 |
| Efficacy of integrated three-dimensional electroanatomic mapping with preacquired magnetic resonance images guide catheter atrial fibrillation ablation | 2007 | Full text not available | <https://pubmed.ncbi.nlm.nih.gov/18269825/> |
| The efficacy of inducibility and circumferential ablation with pulmonary vein isolation in patients with paroxysmal atrial fibrillation. | 2007 | Wrong intervention - circumferential ablation | https://dx.doi.org/10.1111/j.1540-8167.2007.00823.x |
| Does electrogram guided substrate ablation add to the success of pulmonary vein isolation in patients with paroxysmal atrial fibrillation? A prospective, randomized study. | 2009 | Wrong intervention - ablation of complex fractionated atrial electrograms | https://dx.doi.org/10.1111/j.1540-8167.2008.01379.x |
| Ablation of atrial fibrillation: does the addition of three-dimensional magnetic resonance imaging of the left atrium to electroanatomic mapping improve the clinical outcome?: a randomized comparison of Carto-Merge vs. Carto-XP three-dimensional mapping | 2010 | Wrong intervention - pulmonary veins plus linear lesions | https://dx.doi.org/10.1093/europace/euq107 |
| Substrate and Trigger Ablation for Reduction of Atrial Fibrillation (STAR AF): a randomized, multicentre, international trial. | 2010 | Wrong intervention - ablation of complex fractionated electrograms | https://dx.doi.org/10.1093/eurheartj/ehq041 |
| Catheter ablation of persistent atrial fibrillation: anatomically based circumferential pulmonary vein ablation in combination with a potential-guided segmental approach to achieve complete pulmonary vein isolation. | 2011 | Wrong intervention - pulmonary vein isolation and ablation of linear lesions at the roof of the left atrium | https://dx.doi.org/10.1007/s10840-010-9533-1 |
| A prospective, randomized comparison of modified pulmonary vein isolation versus conventional pulmonary vein isolation in patients with paroxysmal atrial fibrillation. | 2012 | Wrong intervention - ablation of high-frequency AF nests | https://dx.doi.org/10.1111/j.1540-8167.2012.02379.x |
| Randomized ablation strategies for the treatment of persistent atrial fibrillation: RASTA study. | 2012 | Wrong intervention - no fibrosis-guided ablation arm | https://dx.doi.org/10.1161/CIRCEP.111.966226 |
| Lesion formation after pulmonary vein isolation using the advance cryoballoon and the standard cryoballoon: lessons learned from late gadolinium enhancement magnetic resonance imaging. | 2015 | Wrong intervention - no fibrosis-guided ablation arm | https://dx.doi.org/10.1093/europace/euu260 |
| Catheter Ablation of Nonparoxysmal Atrial Fibrillation Using Electrophysiologically Guided Substrate Modification During Sinus Rhythm After Pulmonary Vein Isolation. | 2016 | Wrong study design - not an RCT | https://dx.doi.org/10.1161/CIRCEP.115.003382 |
| Spatial Relationship of Focal Impulses, Rotors and Low Voltage Zones in Patients With Persistent Atrial Fibrillation. | 2016 | Wrong study design - not an RCT | https://dx.doi.org/10.1111/jce.12913 |
| Impact of Voltage Mapping to Guide Whether to Perform Ablation of the Posterior Wall in Patients With Persistent Atrial Fibrillation. | 2016 | Wrong study design - not an RCT | https://dx.doi.org/10.1111/jce.12830 |
| Stand-Alone Pulmonary Vein Isolation Versus Pulmonary Vein Isolation With Additional Substrate Modification as Index Ablation Procedures in Patients With Persistent and Long-Standing Persistent Atrial Fibrillation: The Randomized Alster-Lost-AF Trial (Abl | 2017 | Wrong intervention - stepwise approach of PVI followed by complex fractionated atrial electrogram ablation and linear ablation | https://dx.doi.org/10.1161/CIRCEP.117.005114 |
| Clinical Outcome of Electrophysiologically Guided Ablation for Nonparoxysmal Atrial Fibrillation Using a Novel Real-Time 3-Dimensional Mapping Technique: Results From a Prospective Randomized Trial. | 2018 | Wrong intervention - ablation of repetitive-regular activities | https://dx.doi.org/10.1161/CIRCEP.117.005904 |
| Efficacy of an Adjunctive Electrophysiological Test-Guided Left Atrial Posterior Wall Isolation in Persistent Atrial Fibrillation Without a Left Atrial Low-Voltage Area. | 2020 | Wrong intervention - ablation of posterior wall isolation | https://dx.doi.org/10.1161/CIRCEP.119.008191 |
| Circumferential pulmonary vein antrum ablation for the treatment of paroxysmal atrial fibrillation: A randomized controlled trial. | 2020 | Wrong intervention - antrum modification | https://dx.doi.org/10.1111/pace.13863 |
| Low-Voltage-Area Ablation in Paroxysmal Atrial Fibrillation - Extended Follow-up Results of the VOLCANO Trial. | 2022 | Extended results of an existing trial | https://dx.doi.org/10.1253/circj.CJ-21-0476 |
| Single procedural outcomes in the setting of percutaneous ablation for persistent atrial fibrillation: a propensity-matched score comparison between different strategies. | 2022 | Wrong study design - not an RCT of fibrosis-guided ablation | https://dx.doi.org/10.1007/s10840-021-00968-2 |
| Impact of Posterior Left Atrial Voltage on Ablation Outcomes in Persistent Atrial Fibrillation: CAPLA Substudy. | 2023 | Wrong study design - not an RCT of fibrosis-guided ablation | https://dx.doi.org/10.1016/j.jacep.2023.08.002 |
| Catheter Ablation With Morphologic Repetitiveness Mapping for Persistent Atrial Fibrillation. | 2023 | Wrong intervention - periodicity and similarity mapping | https://dx.doi.org/10.1001/jamanetworkopen.2023.44535 |
| Multi-centre, prospective randomized comparison of three different substrate ablation strategies for persistent atrial fibrillation. | 2023 | Wrong intervention - anatomy and electrogram ablation | https://dx.doi.org/10.1093/europace/euad090 |
| Ablation STrategies for Repeat PrOcedures in Atrial Fibrillation Recurrences despite Durable Pulmonary Vein Isolation | 2024 | No comparator of interest - empirical left atrial appendage isolation | https://dx.doi.org/10.1101/2024.04.09.24305585 |
| Extensive ablation for persistent atrial fibrillation patients with mitral regurgitation: Insights from the EARNEST-PVI prospective randomized trial | 2024 | Wrong intervention - ablation strategy with linear and/or CFAE ablation | https://dx.doi.org/10.1016/j.ijcard.2024.132231 |
| Clinical Outcomes of Computational Virtual Mapping-Guided Catheter Ablation in Patients With Persistent Atrial Fibrillation: A Multicenter Prospective Randomized Clinical Trial | 2021 | Wrong intervention - ablation of dominant frequency | https://dx.doi.org/10.3389/fcvm.2021.772665 |
| Baseline left atrial low-voltage area predicts recurrence after pulmonary vein isolation: WAVE-MAP AF results | 2023 | Wrong study design - not an RCT | https://dx.doi.org/10.1093/europace/euad194 |
| MRI-Guided Fibrosis Ablation vs Conventional Catheter Ablation for Patients with Persistent Atrial Fibrillation | 2022 | Wrong study design - not an RCT | https://dx.doi.org/10.1001/jama.2022.16292 |
| Clinical Usefulness of Virtual Ablation Guided Catheter Ablation of Atrial Fibrillation Targeting Restitution Parameter-Guided Catheter Ablation: CUVIA-REGAB Prospective Randomized Study | 2022 | Wrong intervention - targeting high maximal slope of the action potential duration restitution curve | https://dx.doi.org/10.4070/KCJ.2022.0113 |
| Effects of additional ablation of low-voltage areas after Box isolation for persistent atrial fibrillation | 2019 | No comparator of interest - use of BOX isolation | https://dx.doi.org/10.1002/joa3.12169 |
| Diabetes mellitus and atrial remodelling in patients with paroxysmal atrial fibrillation: Role of electroanatomical mapping and catheter ablation | 2018 | Wrong intervention - ablation of complex fractionated atrial electrograms | https://dx.doi.org/10.1177/1479164117752492 |
| Benefits of Atrial Substrate Modification Guided by Electrogram Similarity and Phase Mapping Techniques to Eliminate Rotors and Focal Sources Versus Conventional Defragmentation in Persistent Atrial Fibrillation | 2016 | Wrong intervention - ablation guided by nonlinear phase mapping technique | https://dx.doi.org/10.1016/j.jacep.2016.08.005 |
| A prospective, multicenter evaluation of ablating complex fractionated electrograms (CFEs) during atrial fibrillation (AF) identified by an automated mapping algorithm: Acute effects on AF and efficacy as an adjuvant strategy | 2008 | Wrong intervention - ablation of complex fractionated atrial electrograms | https://dx.doi.org/10.1016/j.hrthm.2007.09.027 |

**Supplementary Table 4: Summary of Findings**

| **Outcome** | **Effect Size**  **95%CI**  **P** | **Studies**  **Sample size** | **NNT to prevent one event or harm** | **Heterogeneity**  **Risk of Bias Assessment** | **Indirectness**  **Imprecision**  **Publication Bias** | **Interpretation**  **Quality of Evidence/GRADE** |
| --- | --- | --- | --- | --- | --- | --- |
| Freedom from atrial arrhythmia relapse | RR= 1.13  1.04-1.23  P=0.004 | 12 RCTs,  2,986 patients | 13.1 patients benefit  67.3% vs. 59.7% | Low heterogeneity (I^2^=30%)  RoB – ↓1 level (Performance) | No indirectness  No Imprecision  No Publication Bias | Significant reduction in atrial arrhythmia relapse with fibrosis-guided ablation  Moderate Quality  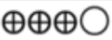 |
| Need for repeat ablation procedure | RR=0.72  0.56-0.92  P=0.023 | 6 RCTs  992 patients | 20.8 patients benefit  12.1% vs. 16.9% | Low heterogeneity (I^2^=0%)  RoB – ↓1 level (Performance) | No indirectness  Imprecision ↓1 level  N.A. (<10 RCT) | Significant reduction in need for repeat ablation procedure  Low Quality  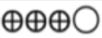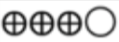 |
| Recurrence of atrial tachycardia | RR=0.75  0.37-1.55  P=0.38 | 7 RCTs  1776 patients | 124.0 patients  benefit  7.4% vs. 8.5% | High heterogeneity (I^2^=60%)  RoB – ↓1 level (Performance) | No indirectness  Imprecision ↓1 level  Publication Bias | No difference in atrial tachycardia relapse  Very Low Quality  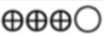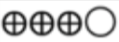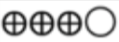 |
| Recurrence of atrial flutter | RR=1.22  0.35-4.28  P=0.29 | 2 RCTs  970 patients | 70.7 patients  harm  7.8% vs. 6.4% | Low heterogeneity (I^2^=0%)  RoB – ↓1 level (Performance) | No indirectness  Imprecision ↓1 level  Publication Bias | No difference in atrial flutter relapse  Low Quality  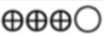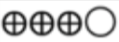 |
| Time to first arrhythmia recurrence | HR=0.79  0.67-0.94  P=0.008 | 6 RCTs  1,528 patients | - | Low heterogeneity (I^2^=19%)  RoB – ↓1 level (Performance) | No indirectness  Imprecision ↓1 level  No Publication Bias | Significant reduction in time to first arrhythmia recurrence  Low Quality  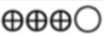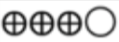 |
| Total procedure time | SMD=-0.47  -2.33-1.39  P=0.58 | 9 RCTs  994 patients | - | High heterogeneity (I^2^=97%)  RoB – ↓1 level (Performance) | No indirectness  Imprecision ↓1 level  N.A. (<10 RCT) | No difference in total procedure time  Very Low Quality  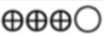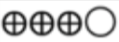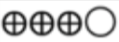 |
| Ablation time | SMD=0.34  -0.66-1.34  P=0.40 | 5 RCTs  829 patients | - | High heterogeneity (I^2^=93%)  RoB – ↓1 level (Performance) | No indirectness  Imprecision ↓1 level  N.A. (<10 RCT) | No difference in ablation time  Low Quality  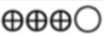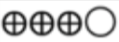 |
| All periprocedural complications | RR=1.44  0.82-2.55  P=0.18 | 10 RCTs  2,867 patients | 50.7 patients harm  4.4% vs. 2.8% | Low heterogeneity (I^2^=30%)  RoB – ↓1 level (Selection) | No indirectness  Imprecision ↓1 level  No Publication Bias | No difference in all periprocedural complications  Low Quality  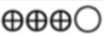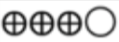 |
| Major periprocedural complications | RR=1.16  0.58-2.31  P=0.62 | 8 RCTs  2,403 patients | 176.8 patients harm  3.0% vs 2.5% | Low heterogeneity (I^2^=20%)  RoB – ↓1 level  (Performance) | No indirectness  Imprecision ↓1 level  N.A. (<10 RCT) | No difference in major periprocedural complications  Low Quality  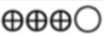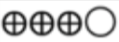 |

**Supplementary Table 5: Pooled results for recurrence of atrial tachycardia and atrial flutter**

|  | **Number of studies** | **Number of patients** | **Rate in fibrosis-guided ablation arm** | **Rate in control arm** | **Pooled risk ratio (95% confidence interval)** | **P value** | **I^2^ value** |
| --- | --- | --- | --- | --- | --- | --- | --- |
| **Recurrence of Atrial tachycardia** | 7 | 1776 | 68/889 (7.4%) | 75/887 (8.5%) | 0.75 (0.37–1.55) | 0.38 | 60% |
| **Recurrence of Atrial flutter** | 2 | 970 | 38/486 (7.8%) | 31/484 (6.4%) | 1.22 (0.35–4.28) | 0.29 | 0% |

**Supplementary Figure 1: Risk of Bias using ROB 1.0 tool**

**
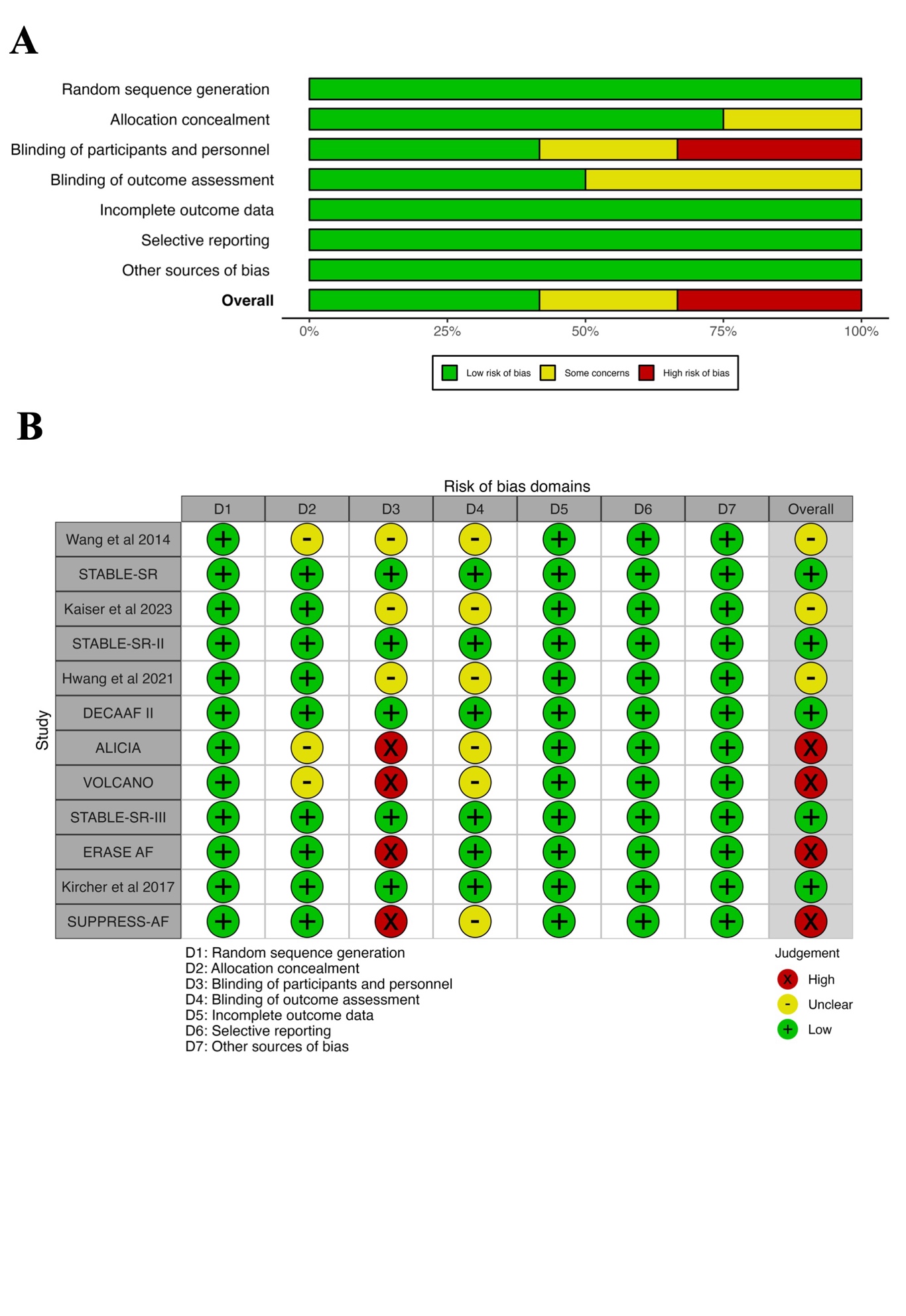
**

**Justification:**

**Wang et al 2014** do not provide information on allocation concealment or the blinding of participant. Blinding during outcome assessment is also not mentioned. Since outcomes could be subjectively assessed, the unclear blinding introduces potential bias.

**Kaiser et al 2023** describe a randomized approach, a 1:1 ratio, for assigning patients to either PVI with LVA ablation or PVI with additional ablation. However, there is no mention of blinding for patients or personnel. The document also lacks details on whether outcome assessors were blinded.

**Hwang et al 2023** do not specify if blinding was done for participants, personnel, or outcome assessors.

**ALICIA** lacks details on allocation concealment, such as whether the randomization sequence was hidden from investigators. Conducted as an open-label trial, it did not include blinding for participants or personnel, which presents a high risk. There is an unclear indication if outcome assessors were blinded to treatment allocation, increasing the potential for bias.

**VOLCANO** does not specify allocation concealment methods, such as whether the assignment was concealed from enrolling clinicians. As an open-label trial, the lack of blinding may have allowed knowledge of intervention group assignments to influence outcomes. The absence of clear blinding for outcome assessment also raises concerns.

**ERASE-AF** was conducted as an open-label study without blinding participants or personnel. However, outcome adjudication was handled by a core lab team blinded to treatment assignment, likely reducing detection bias in the objective assessment of primary and secondary efficacy endpoints.

**SUPPRESS-AF** was also an open-label trial without participant or personnel blinding. Nevertheless, outcome events, including arrhythmia recurrence, were adjudicated by an endpoint committee separate from the trial’s developers and funders but blinding details remain unclear.

**Supplementary Figure 2: Publication bias assessment**

**A. atrial arrhythmia relapse**

**
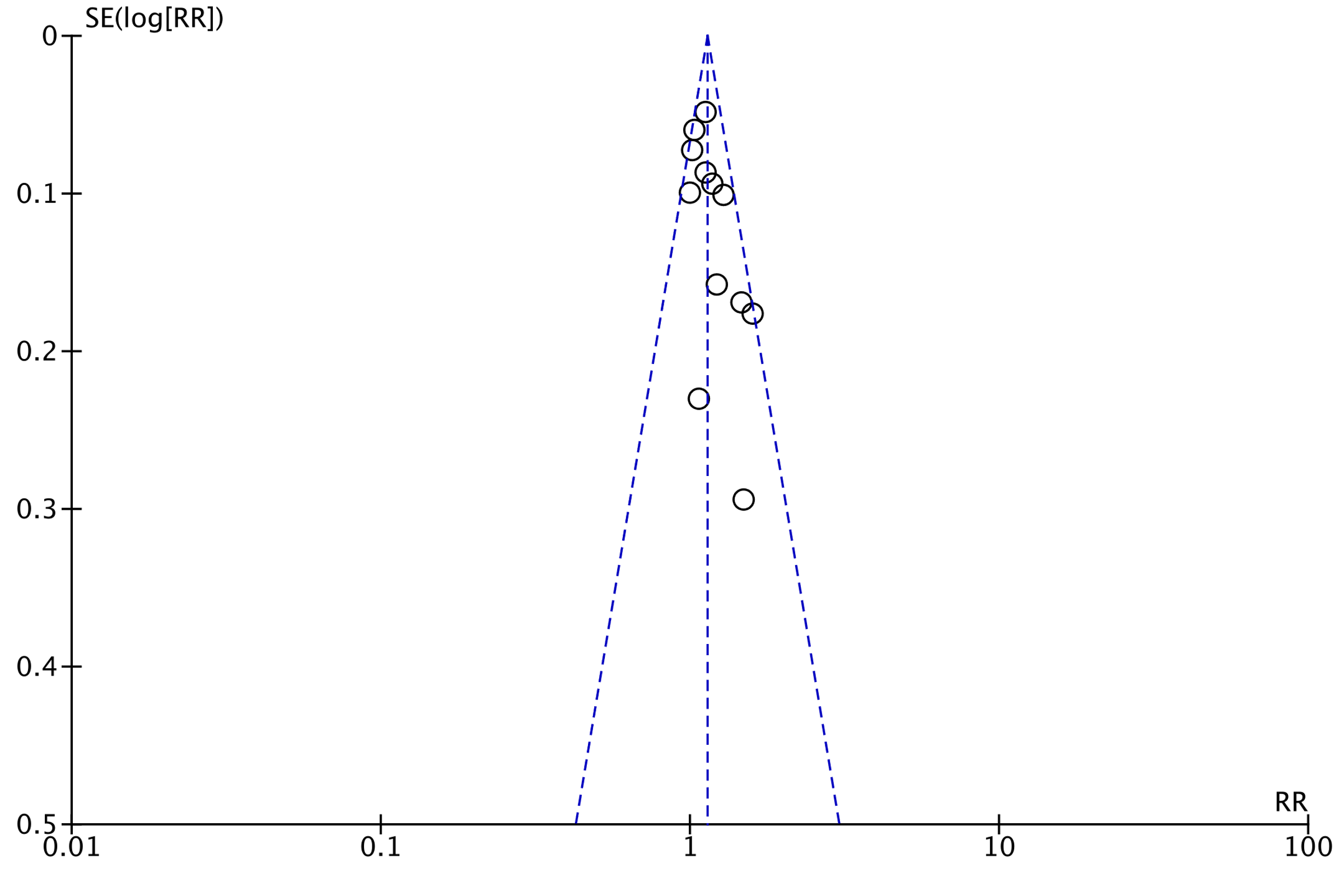
**

**B – procedural complications**

**
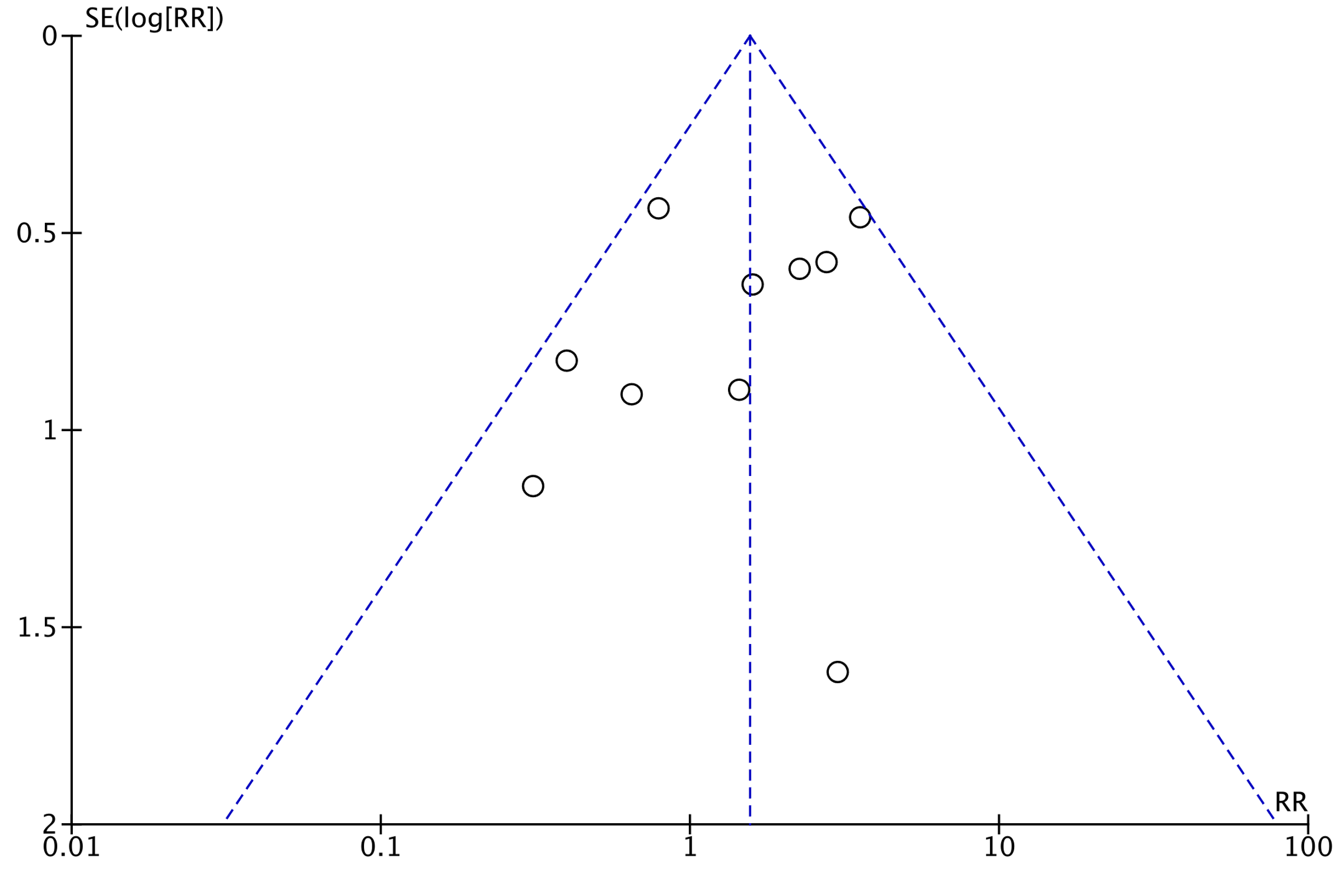
**

**Supplementary Figure 3: Freedom from atrial arrhythmias among patients with baseline mapped fibrosis**

**
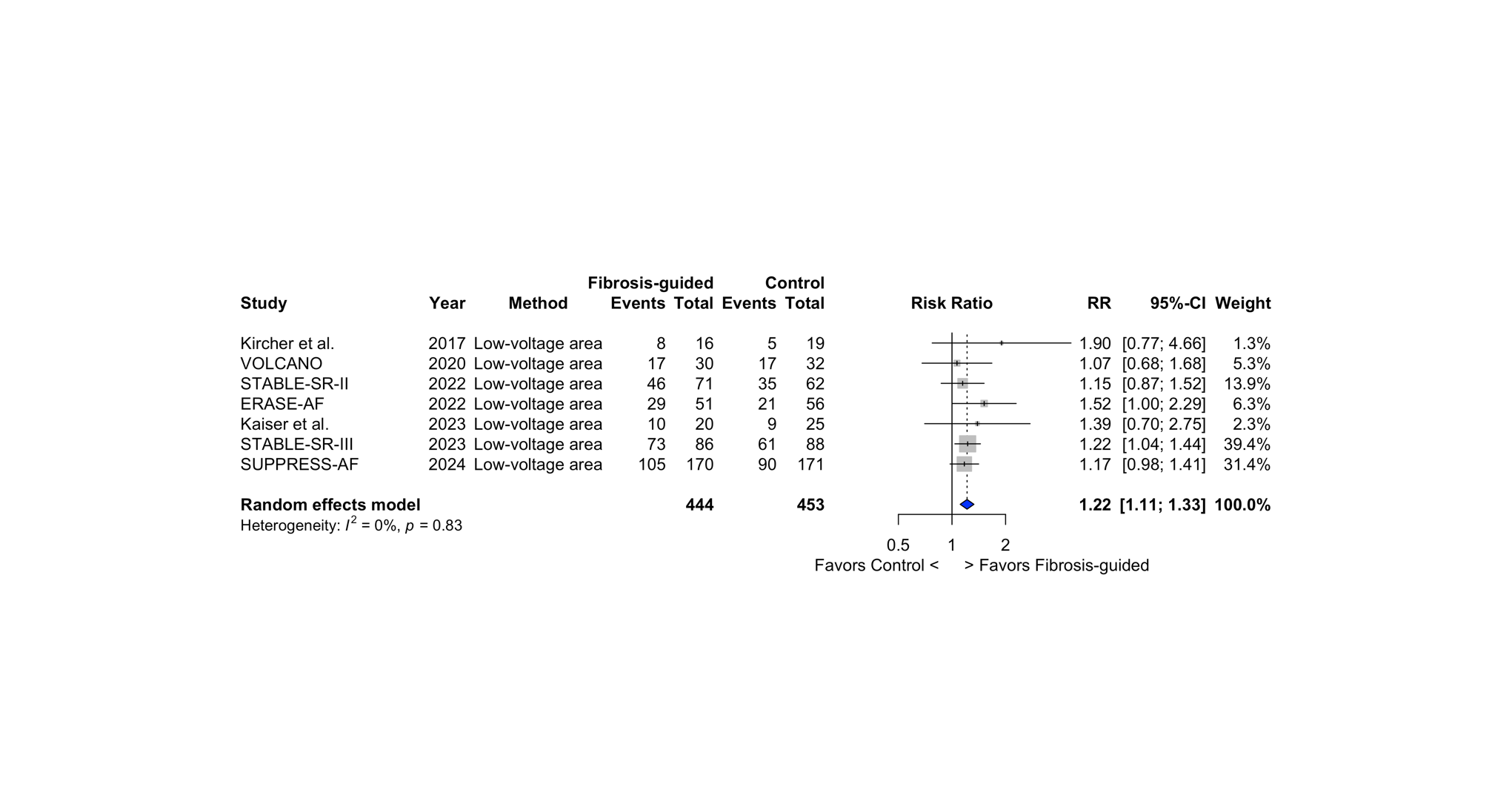
**


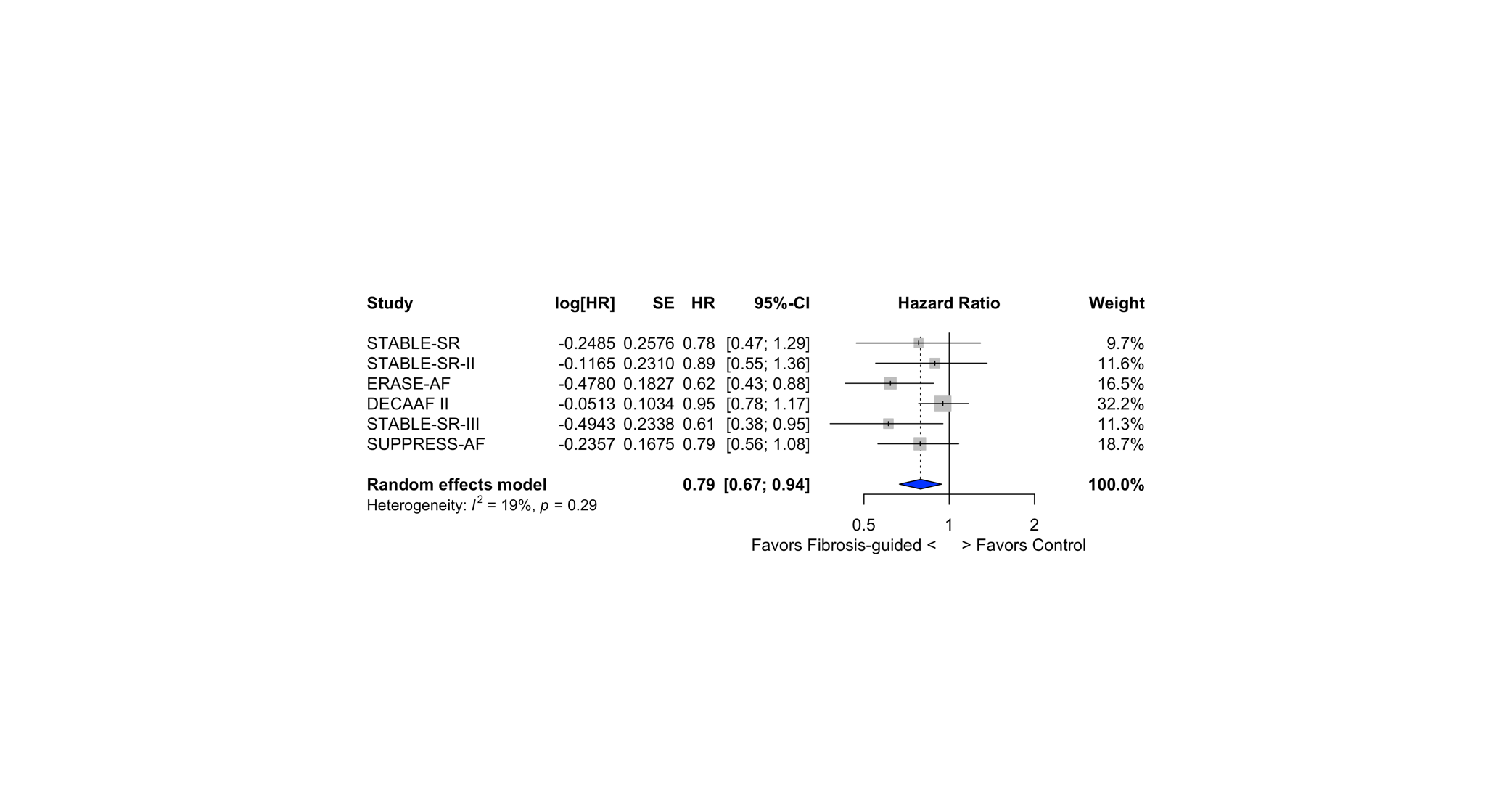
**Supplementary Figure 4: Time-to-event analysis for atrial arrhythmia relapse**

**
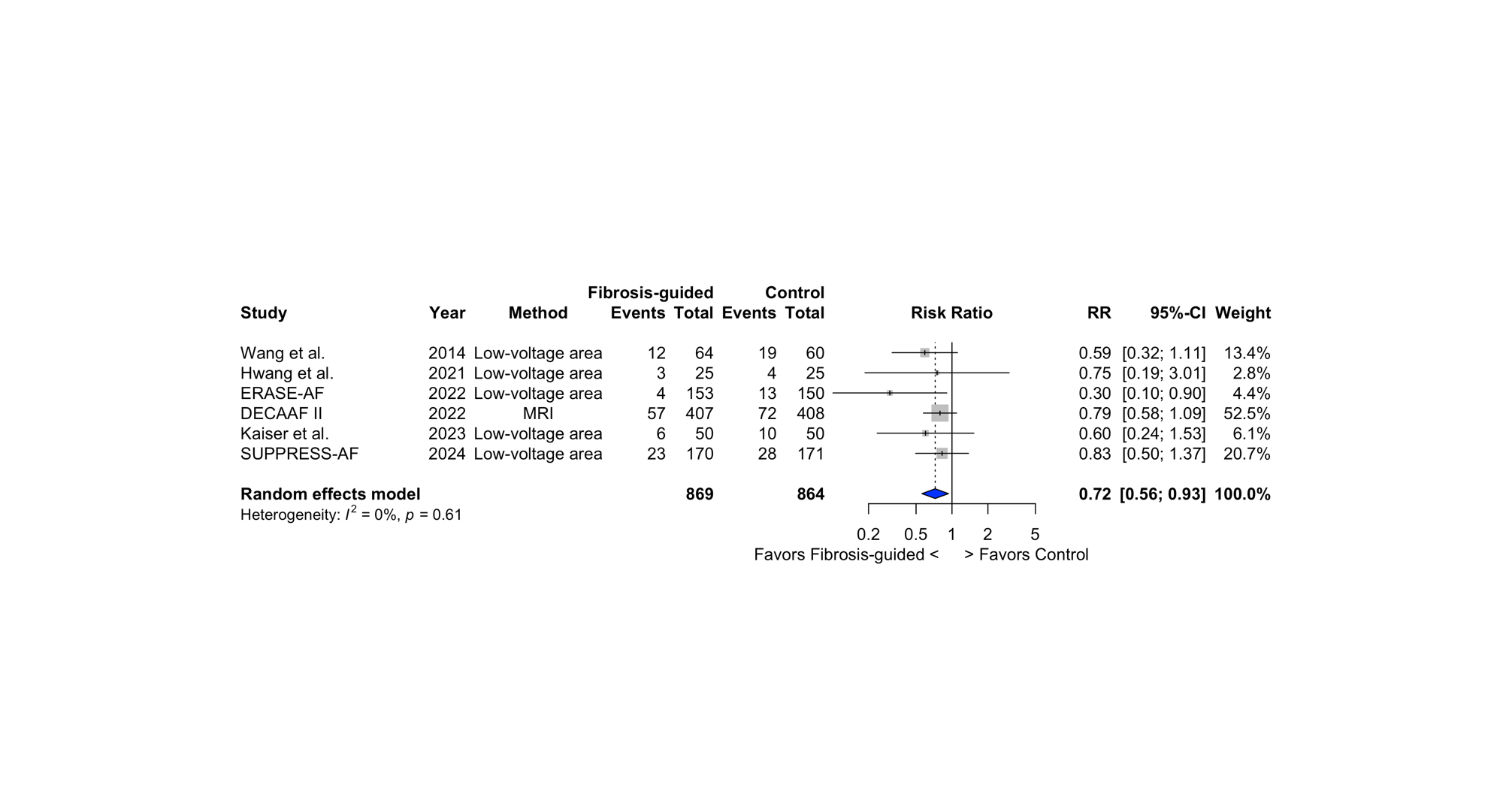
Supplementary Figure 5: Rate of repeat ablation procedure**

**
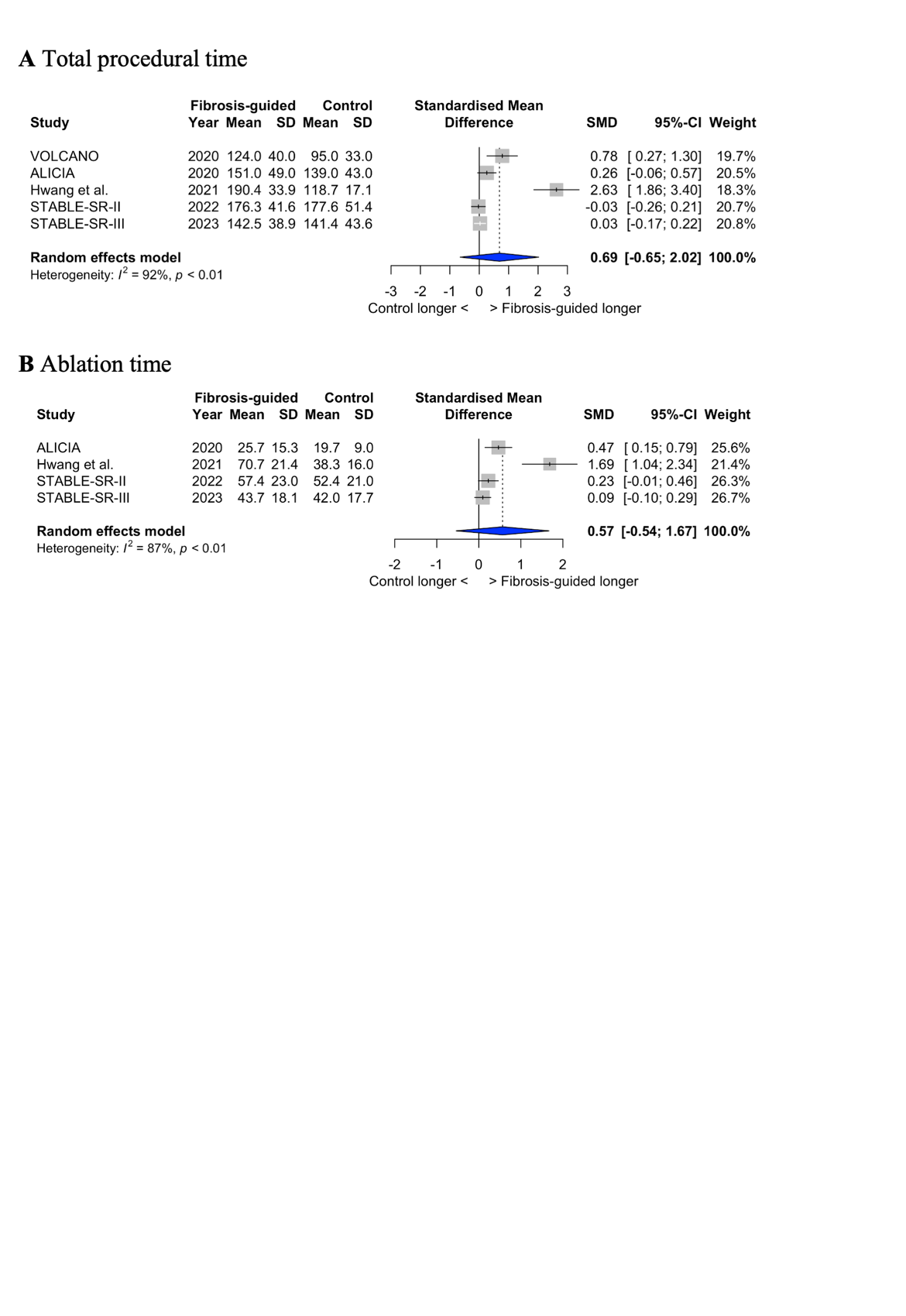
Supplementary Figure 6: Total procedural and ablation time for studies with PVI only as the control arm**

**
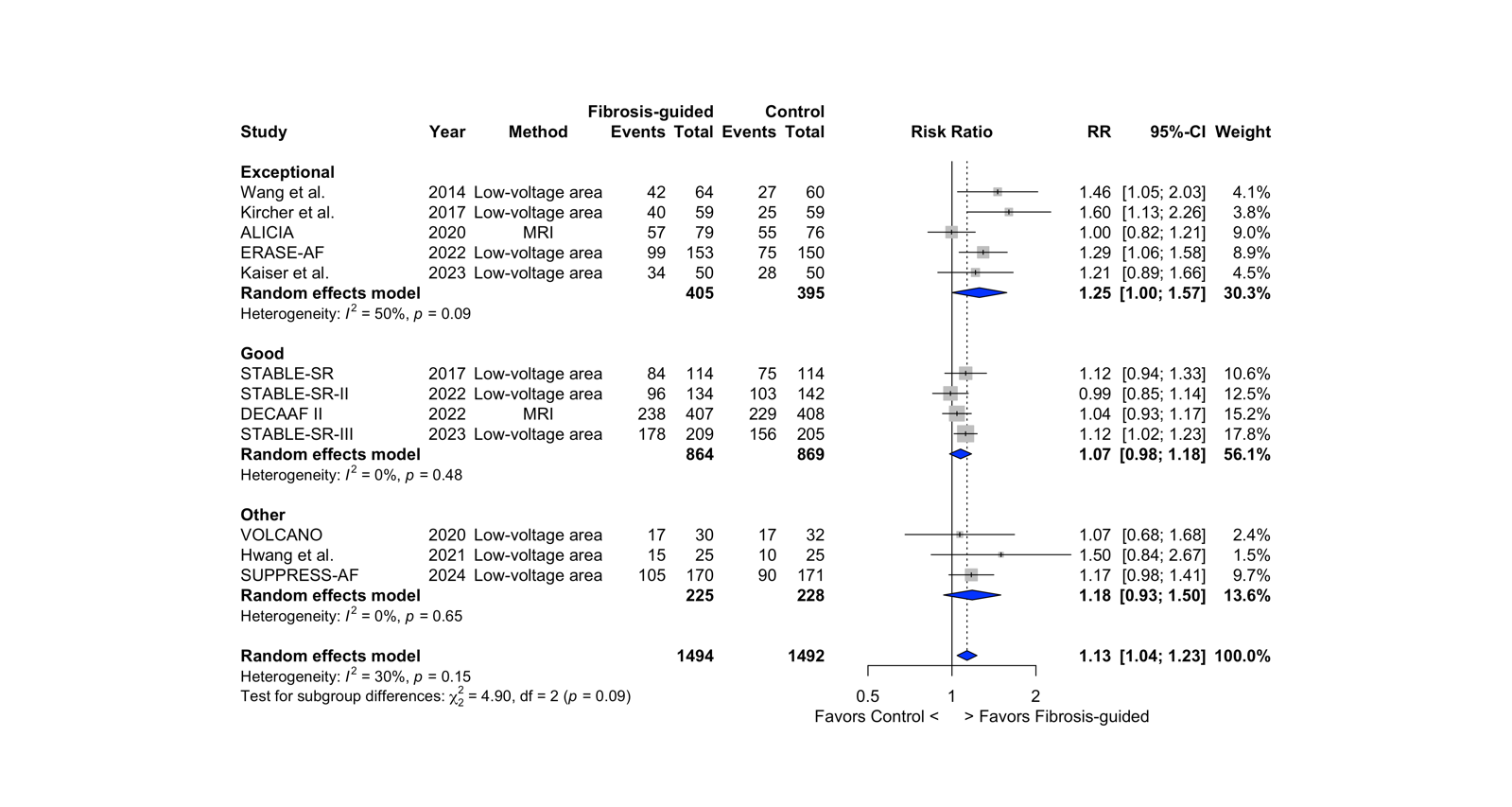
Supplementary Figure 7: Freedom from atrial arrhythmias based on quality of ablation technique**

**Supplementary Figure 8: Freedom from atrial arrhythmias based on the control group ablation method**

**
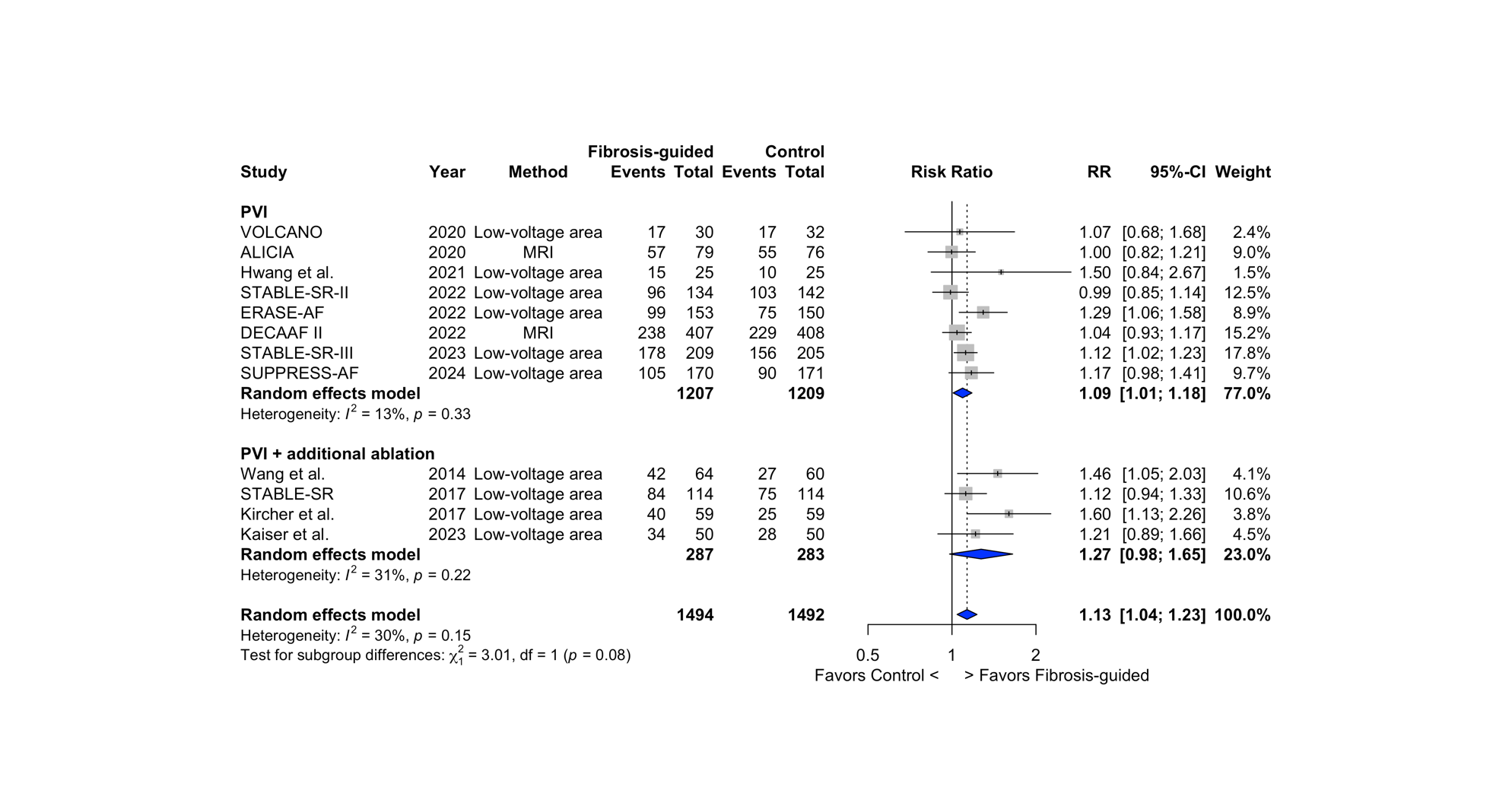
**

**Supplementary Figure 9: Freedom from atrial arrhythmias based on the low-voltage area threshold**

**
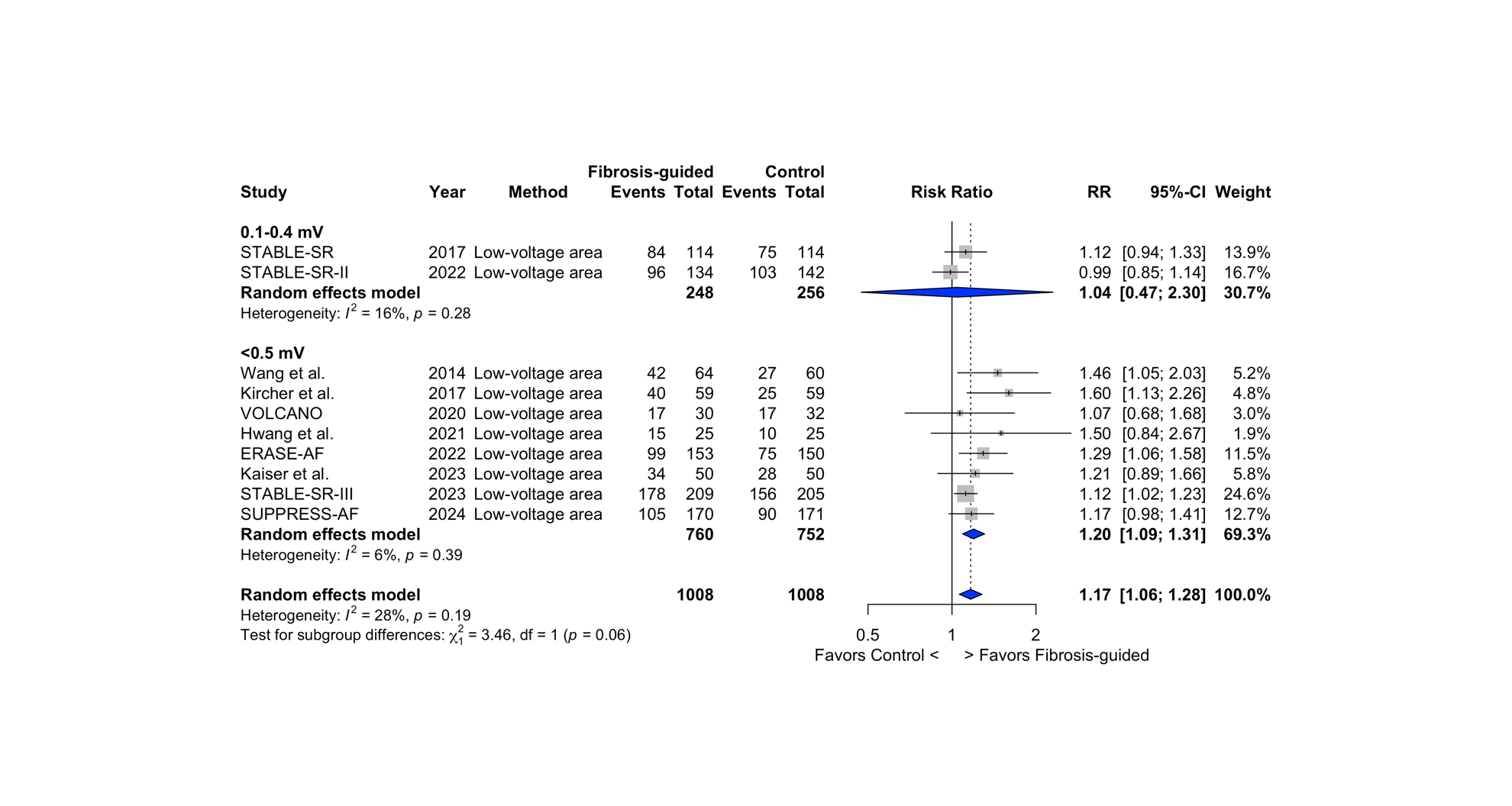
**

**
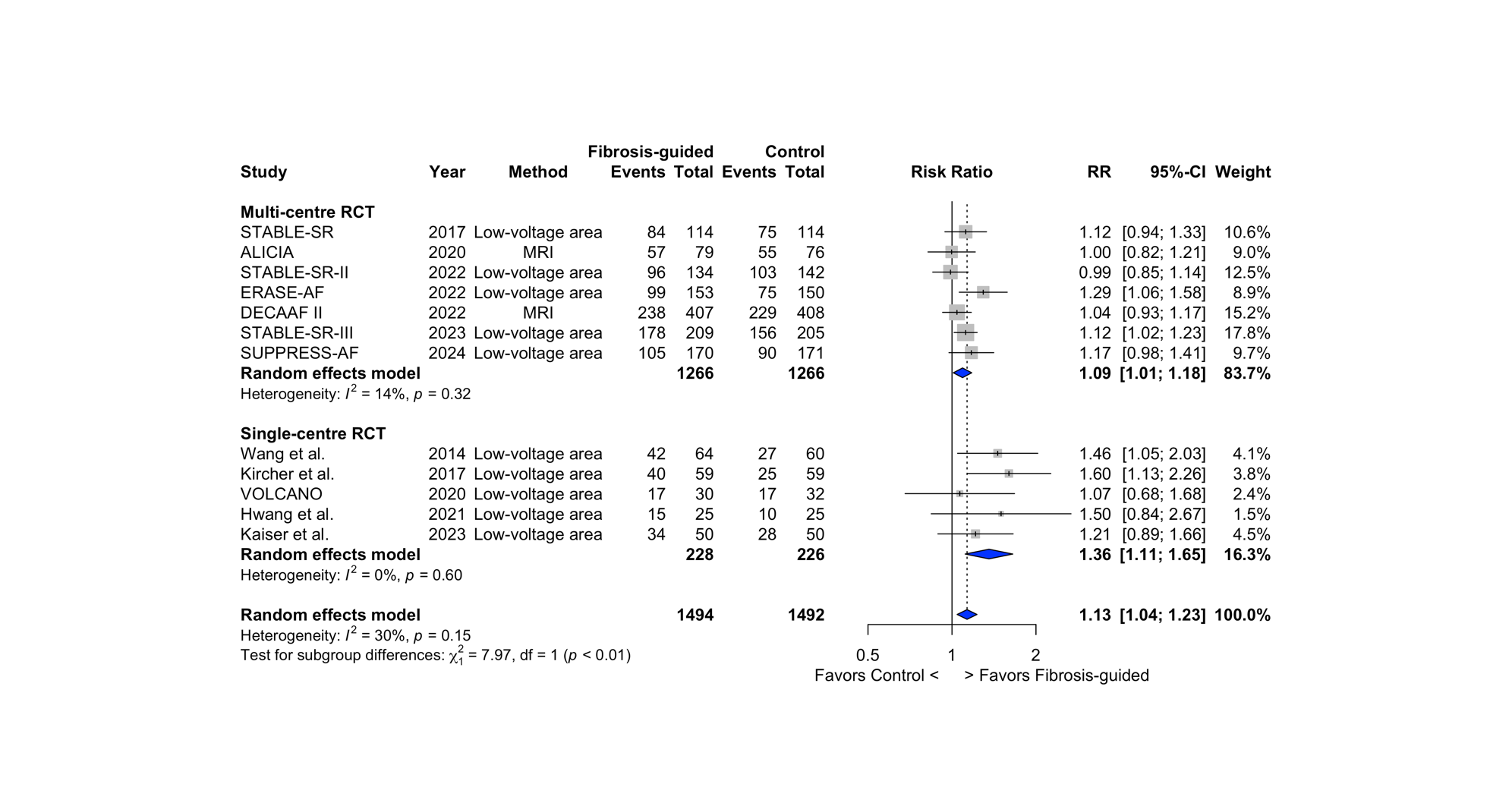
Supplementary Figure 10: Freedom from atrial arrhythmias based on trial design**

**
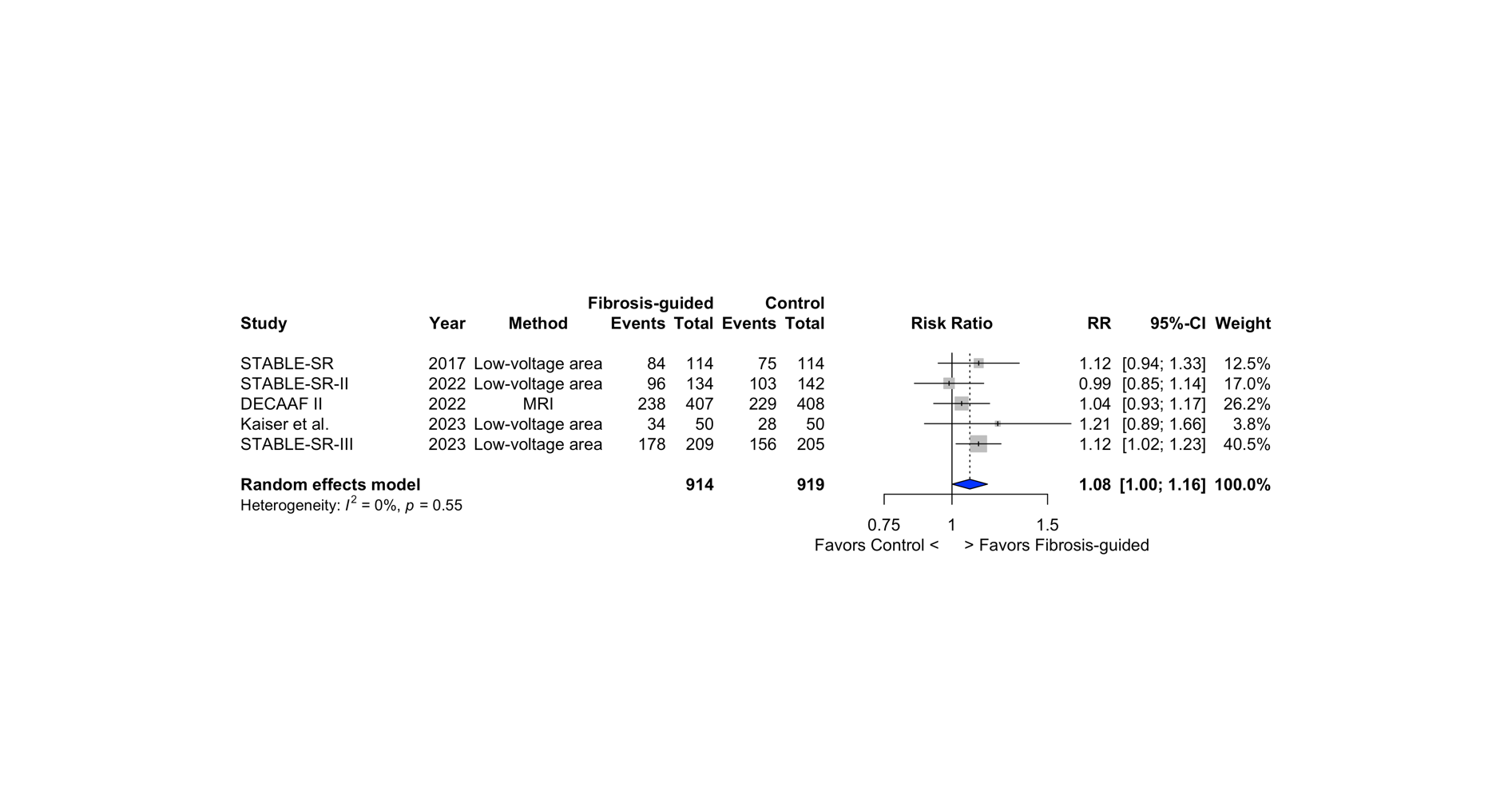
Supplementary Figure 11: Freedom from atrial arrhythmias of low-bias studies**

**Supplementary Figure 12: Freedom from atrial arrhythmias based on study year**

**
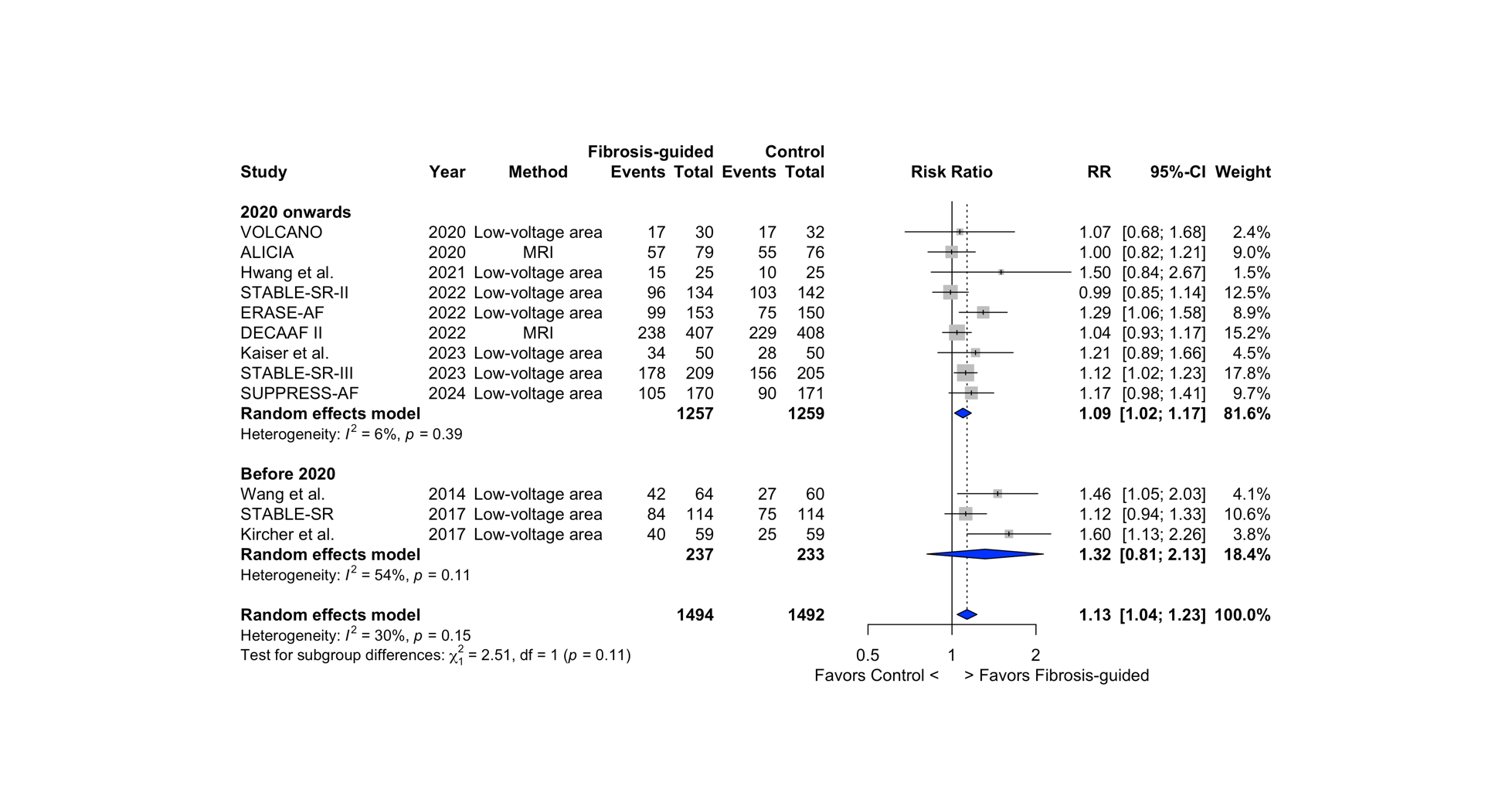
**


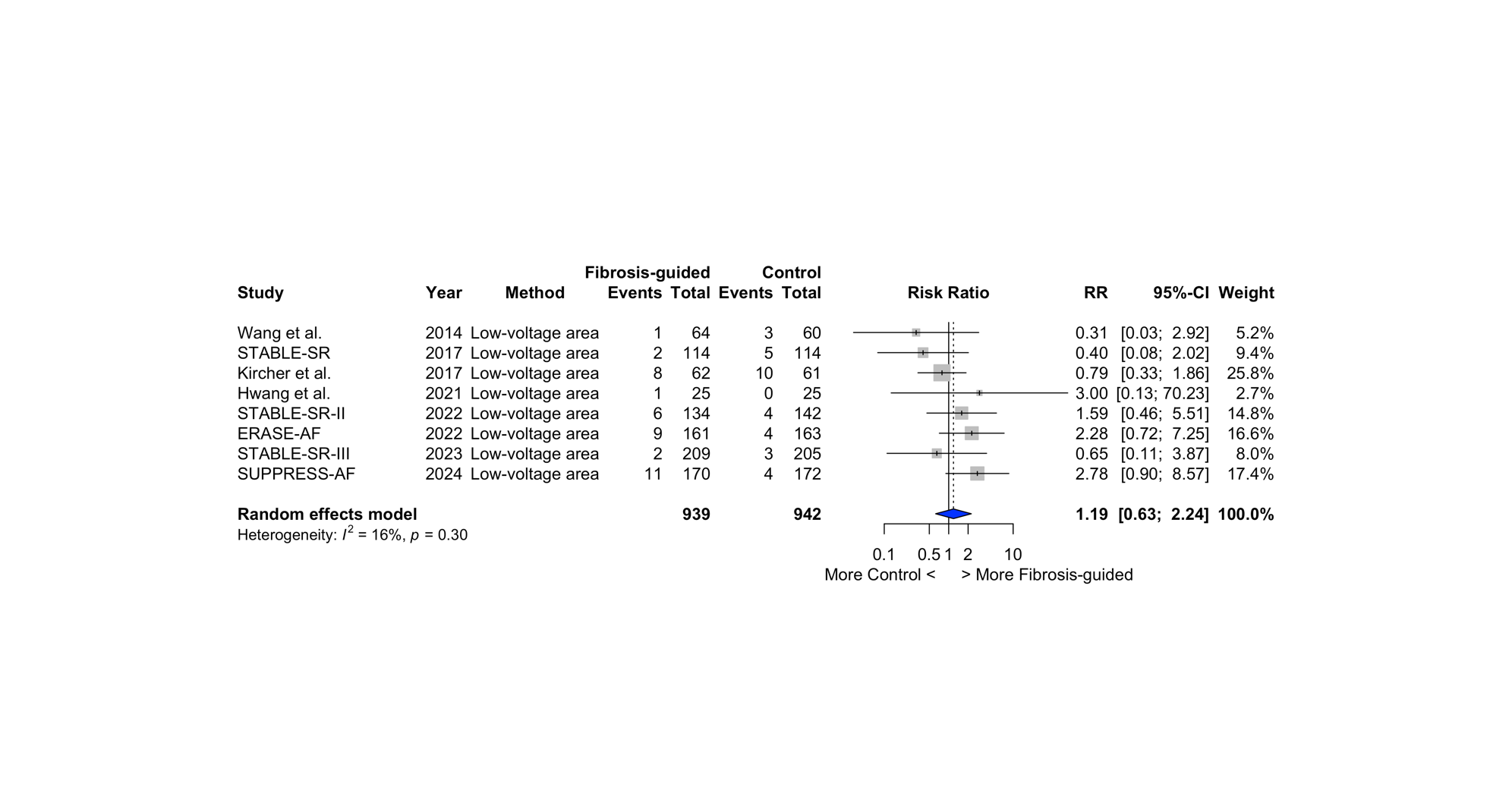
**Supplementary Figure 12: Periprocedural complications for LVA ablation studies only**
